# A Self-Assembling NHC-Pd-Loaded Calixarene as a Potent Catalyst for the Suzuki-Miyaura Cross-Coupling Reaction in Water

**DOI:** 10.3390/molecules25061459

**Published:** 2020-03-24

**Authors:** Arnaud Peramo, Ibrahim Abdellah, Shannon Pecnard, Julie Mougin, Cyril Martini, Patrick Couvreur, Vincent Huc, Didier Desmaële

**Affiliations:** 1 Institut Galien Paris-Sud, CNRS UMR 8612, Université Paris-Saclay, Faculté de Pharmacie, 5 rue JB Clément, 92296 Châtenay-Malabry, France; arnaud.peramo@gmail.com (A.P.); shannon.pecnard@u-psud.fr (S.P.); julie.mougin@u-psud.fr (J.M.); patrick.couvreur@u-psud.fr (P.C.); 2 Institut de Chimie Moléculaire et des Matériaux d’Orsay, CNRS UMR 8182, Université Paris Saclay, Bâtiment 420, 91405 Orsay, France; ibrahim_abdellah@hotmail.com (I.A.); cyril.martini@novecal.com (C.M.); 3 NOVECAL, 86 rue de Paris, 91400 Orsay, France

**Keywords:** NHC, nanoparticle, calixarene, palladium catalyst, Suzuki-Miyaura reaction, amino-acids, water

## Abstract

Nanoformulated calix[8]arenes functionalized with *N*-heterocyclic carbene (NHC)-palladium complexes were found to be efficient nano-reactors for Suzuki-Miyaura cross-coupling reactions of water soluble iodo- and bromoaryl compounds with cyclic triol arylborates at low temperature in water without any organic co-solvent. Combined with an improved one-step synthesis of triol arylborates from boronic acid, this remarkably efficient new tool provided a variety of 4′-arylated phenylalanines and tyrosines in good yields at low catalyst loading with a wide functional group tolerance.

## 1. Introduction

**Figure 1:**
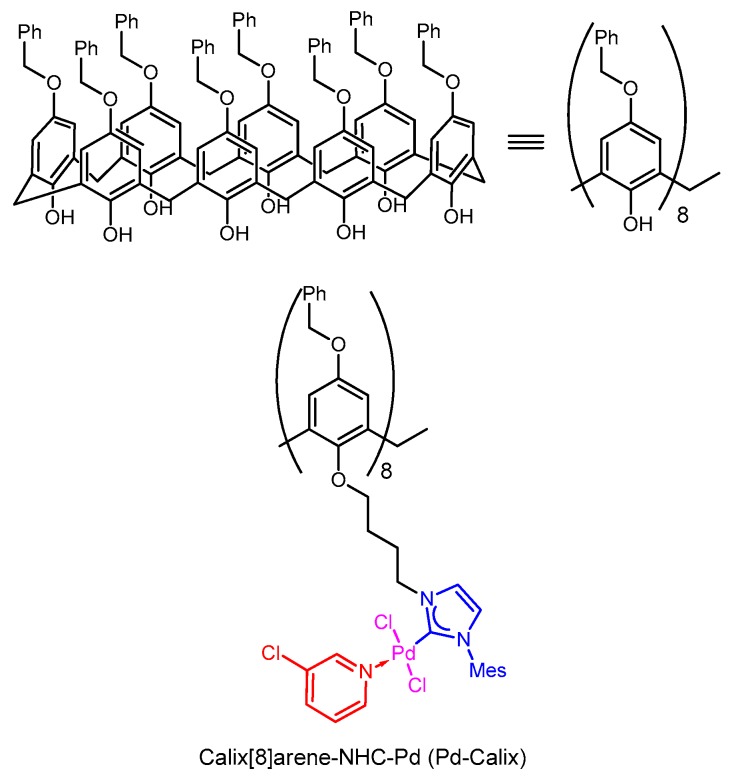
General structure of calix [8]arenes (top) and structure of the calix[8]arene bearing eight NHC-palladium units (Pd-Calix) (bottom). Mes = Mesityl.

The Suzuki-Miyaura reaction has emerged as one of the most powerful tool for C–C bond formation because of its compatibility with a broad range of functional groups giving rise to numerous applications throughout organic chemistry, synthesis of pharmaceutical compounds [1,2,3] and materials science [4,5]. Aqueous Suzuki-Miyaura cross-coupling reactions are particularly promising due to the water tolerance of boronic acids compared with the organometallic reagents required for other cross-coupling protocols. As a consequence, the Suzuki-Miyaura reaction has been widely developed under homogenous or heterogenous catalytic conditions in water media and a large array of different ligands and palladium complexes including micellar catalysis conditions nanoparticulate formulations have been designed to increase efficiency under aqueous conditions [6,7,8,9,10,11,12,13,14,15,16]. The water and functional group tolerance of the Suzuki-Miyaura reaction culminated in the cross-coupling of iodoarylated proteins under physiological conditions opening the way to a realm of chemical-biology applications [17,18,19,20]. Reactions in water are not restricted to those with biomolecules: due to water’s ideal proprieties (abundance, environmental compatibility, non-toxicity and nonflammability) a wide range of catalytic and organic reactions have been developed in water as solvent [21]. In this context, calixarene macromolecules have been used for Suzuki-Miyaura cross-coupling reactions as supports for palladium catalyst. In particular, calix[4]arenes bearing phosphine or N-heterocyclic carbene (NHC) ligands have demonstrated good catalytic efficiencies in this reaction [22,23,24,25,26]. There are a few examples of the use of calix[4]arenes as catalyst supports in mixtures of water and dioxane [22], however, to our knowledge no reactions in pure water have been reported using unsupported calixarene. Recently, the synthesis of a large-cavity calix[8]arene anchoring eight NHC-palladium catalytic heads (Pd-Calix, Figure 1) was reported [27]. This catalyst demonstrated good catalytic properties and low metal leaching for Suzuki-Miyaura cross-coupling reactions with bromoarenes in green solvent such as ethanol, but the Pd-Calix was also tested in water/organic mixtures giving promising results. On the other hand this catalyst was found much less efficient in pure water [27]. Assuming that nanoparticles (NPs) embedding the Pd-Calix would show improved catalytic efficiency over the bulk material, a nanoformulation of this Pd-Calix was realized. Herein we describe the use of Pd-Calix nanosuspension (Pd-Calix-NS) as an efficient catalyst for the Suzuki-Miyaura reaction in aqueous solution at 37 °C. A variety of water-soluble cyclic triolborates were obtained in a one-step process from boronic acids, and their reactions with different haloarenes under Suzuki-Miyaura cross-coupling conditions using the Pd-Calix-NS are described.

## 2. Results and Discussion

The calix [8] arene-NHC-Pd catalyst was synthesized according to the method previously reported [27]. Briefly, chlorobutyl appendages were first bound to the eight free phenol groups of benzyloxycalix[8]arene and the chlorides then displaced with 3-(mesityl)-2,3-dihydro-1*H*-imidazole. The palladium carbene complexes were next generated with palladium chloride in the presence of potassium carbonate and 3-chloropyridine as extra ligand.

Formulation of Pd-Calix was first performed using the nanoprecipitation/solvent evaporation method using THF as organic solvent [28]. Nanoparticles with a 116 nm average hydrodynamic diameter were obtained as revealed by dynamic light scattering (DLS) (Table 1). Unfortunately, this formulation was found quite unstable after few days. To address this problem, the emulsion-solvent evaporation method was attempted using CH_2_Cl_2_ to solubilize the catalyst [29]. The organic phase was poured into an aqueous solution containing sodium cholate (1.5%) as surfactant, the two phases were emulsified by sonication and the solvent was evaporated.

**Figure 2:**
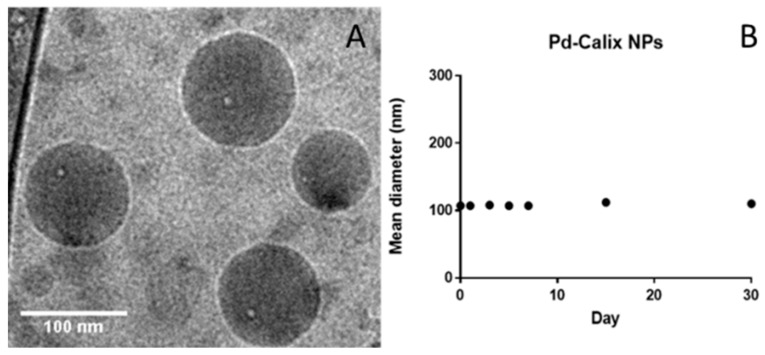
(**A**): Cryo-TEM images of the nanoparticles of the Pd-Calix-NS prepared by emulsion-solvent evaporation; (**B**): Evolution of the mean diameter of the nanoparticles of the Pd-Calix-NS upon incubation at 25 °C in water over one-month.

Monodisperse NPs with a mean diameter of 112 nm (Z average determined by DLS) and a polydispersity index (PdI) of 0.2 were thus obtained. The formulation was found stable upon 30-day storage thanks to their quite negative zeta potential (Table 1). CryoTEM imaging revealed spherical NPs in line with DLS measurements (Figure 2).

Following our early finding that palladium stabilized PLGA-PEG NPs were an efficient catalyst for the Suzuki-Miyaura couplings of 4-iodophenylalanine with phenylboronic acid derivatives, we chose to evaluate the present catalyst in the same model reaction [30]. Thus, reaction of *N*-Boc-4-iodo-l-phenylalanine (**1a**) with phenylboronic acid in the presence of 0.005 mol% of Pd-Calix-NS (0.04 mol% of Pd per mol of substrate) in pH 8.0 phosphate buffer at 37 °C reached 40% conversion into diphenylalanine **3a** after 3 h (Table 2, entry 1).

Although boronic acids are the originally used organoboron derivative for Suzuki-Miyaura reaction, they suffered from some drawbacks such as protodeboronation and oxidation. More nucleophilic organoboron reagents were thus developed with an improved stability and reactivity profile [31]. Among them, potassium phenylfluoroborate [32] and *N*-methyliminodiacetic acid phenylboronates (MIDA) [33] provided very low conversion rate or no reaction. On the other hand, cyclic triol phenylborate [34] turned out to give a full conversion in 3 h using 0.005 mol% of catalyst (0.04 mol% of palladium) per mol of substrate (Table 2, entry 4). Such a superior reactivity of borate salts squared with our previous results with Pd NPs stabilized by PLGA-PEG in water [30]. The hydrolytic stability of the cyclic triol borate derivatives in the reaction conditions (checked by NMR in D_2_O-PBS) must be emphasized and clearly accounted for the efficiency of the process. Interestingly, there was no need of added base to carry out the reaction and the control of the pH was simply achieved using PBS buffer. This feature is of crucial interest to enlarge the process to biomolecules.

To evaluate the influence of the nanoformulation, the bulk catalyst without sodium cholate was used at the same concentration on the same benchmark reaction (Table 2, entry 4). However, less than 5% of conversion was observed highlighting the tremendous impact of the nano-reactor design on the catalysis efficiency. Interestingly, the amount of catalyst can be further diminished to 0.001 mol% on small scale reactions (3–4 mg) without reducing the overall conversion rate, highlighting the remarkable catalytic efficiency of the present nanosuspension. Although similar full conversion was achieved with the Pd-PLGA-PEG NPs catalyst (Entry 6), this formulation suffered of long-term stability issue. On the other hand^,^ the water-soluble Davis catalyst (Entry 7) is very easy to handle but required much higher amount of palladium.

**Figure 3:**
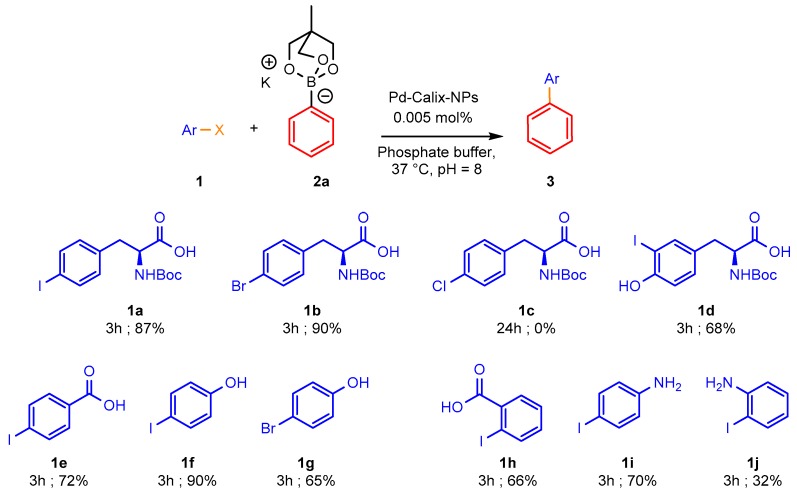
Reactivity of cyclic phenyltriolborate with selected water-soluble aryl halides. Aryl halide (1 equiv.), cyclic triol phenylborate (3 equiv.), Pd-Calix-NS (0.005 mol%), phosphate buffer (20 mM, pH = 8), 37 °C 3 h. Isolated yield after chromatographic purification.

Having identified the best organoboron partner we turned our efforts to expand the scope of the process to other water-soluble aryl halides. The results obtained using various halides with cyclic triol phenylborate **2a** and 0.005 mol% of Pd-Calix-NS as catalyst are summarized in Figure 3. The influence of the halide was briefly investigated. 4-Iodo- and 4-bromophenylalanine underwent reaction with similar high yield. By contrast the chloride derivative **1c** was found completely unreactive. In addition to amino acid derivatives, we investigated several water soluble simple aromatic halides. The presence of carboxylic acid, phenol or amino groups on the arene ring did not affect the reaction, the main limitation being the solubility of the starting material in water. Even 2-iodoaniline underwent the desired coupling albeit at lower rate. 

The influence of the structure of the cyclic triol borate was next explored. Synthesis of cyclic triol borates from boronic acids usually involved initial ester formation with 1,1,1-tris (hydroxymethyl) ethane in toluene with azeotropic removal of water followed by cyclization upon treatment with KOH in the same conditions. We found that the requisite cyclic triol borates can more easily be obtained in a one-step process from boronic acids by a simple stirring with the triol in the presence of 1 equiv. of KOH in dioxane at 30 °C.

The desired compounds were then isolated by simple filtration. This new method was found more convenient that the original procedure and was amenable to large laboratory scale preparation [34]. The bench-stable borate salts were thus obtained in good yields with a variety of aryl and heteroaryl compounds (Table 3). Hindered or electron poor boronic acids required longer reaction time (compounds **2g**, **2h**). Replacement of KOH base by another alkaline earth metal afforded the corresponding metal borate salt in similar yield (compounds **2b**–**d**). On the other hand, the poorly crystallizing tetrabutylammonium cyclic triol borate (**2e**) was obtained in only 20% yield.

With the small library of cyclic triol borate salts in hand, their reaction with *N*-Boc-4-iodo-l-phenylalanine (**1a**) was next examined (Table 4). The screenings of the influence of the counter anion clearly established that potassium and at a lesser extend sodium salts were the most effective. On the other hand the NBu_4_ counter ion was found detrimental to the efficiency of the coupling. It appears that electron withdrawing group on the aromatic ring or steric hindrance made the reaction slower. Nevertheless both, 3′-nitrodiphenylalanine (**3ah**) and [(2′-methylphenyl) phenyl] alanine (**3ag**) were isolated in good yields by prolonging the reaction time to 16 h. Moreover, when the pH was reduced to 7 and 6 the coupling still took place with only a small reduction of yield (Entries **2** and **3**). The latter result may be useful for substrates poorly soluble at basic pH and to conduct reaction with biomolecules at physiological pH [35]. Heterocyclic triolborates, including furan and thiophene rings could also be used affording **3ai**, **3aj** in satisfactory yields. Possibility to recycle the catalyst was briefly investigated. Unfortunately attempts to resuspend the catalyst pellet obtained by centrifugation after the first reaction cycle failed, presumably because it was not possible to adjust the require amount of sodium cholate.

## 3. Experimental

### 3.1. General Information

Infrared (IR) spectra were obtained using a Fourier Transform Bruker Vector 22 spectrometer. Only significant absorptions are listed. The ^1^H and ^13^C NMR were recorded using a Bruker Advance 300 (300 and 75 MHz, respectively) spectrometer. Recognition of methyl, methylene, methine and quaternary carbon nuclei in ^13^C NMR spectra rests on the J-modulated spin-echo sequence. All chemical shifts are quoted on the δ scale in ppm using residual solvent as the internal standard (^1^H NMR: CDCl_3_ = 7.26; D_2_O = 4.79; DMSO-*d*_6_ = 2.50; D_3_COD = 3.33 and ^13^C NMR: DMSO-*d*_6_ = 39.5). Coupling constants (J) are reported in Hz with the following splitting abbreviations: s = singlet, d = doublet, t = triplet, q = quartet, quint = quintet and m = multiplet. ^1^H and ^13^C NMR spectra of compounds **2a, 2e-j, 3a, 3d, 3e-f, 3h-j, 3af, 3ag, 3ah, 3ai, 3aj** are provided in Supplementary Materials. Mass spectra were recorded using an LTQ-Velos Pro Thermofisher Scientific spectrometer. The sizes of the obtained Pd nanoformulations were measured using a Malvern particle size analyzer [nano ZS (173° scattering angle)]. The morphology of the different palladium nanoformulations was examined by transmission electron microscope (TEM) using a JEOL JEM 100CXII transmission electron microscope at an accelerating voltage of 100 kV and for high resolution image using a JEOL JEM 2010 instrument at 200 kV. Deionized water was used for chemical reactions and Milli-Q purified water for nanoparticle preparation. Bidistilled MilliQ water was produced using a water purification system (Millipore). Chemicals were obtained from Carbosynth Limited (UK), Sigma Aldrich Chemical Co (France), Fluorochem and Alfa Aesar (France) and were used without further purification. Solvents of analytical grade were obtained from VWR. Phosphate buffer saline (PBS) pH 7.4 was obtained from Sigma Aldrich. Anhydrous DMF and dioxane were obtained from Sigma Aldrich. Tetrahydrofuran (THF) was distilled from sodium/benzophenone ketyl and CH_2_Cl_2_ from CaH_2_. The calix [8]arene-NHC-Pd catalyst was synthesized according to the method previously reported [27]. All reactions involving air- or water-sensitive compounds were routinely conducted in glassware, which was flame-dried under a positive pressure of nitrogen or argon. Analytical thin-layer chromatography was performed on Merck silica gel 60F254 glass precoated plates (0.25 mm layer). Column chromatography was performed on Merck silica gel 60 (230–400 mesh ASTM (American Standard Test Sieve Series). Diaryl and heteroaryl compounds were chromatographed eluting with AcOEt/cyclohexane mixture 1:4 to 1:1. The 4-substituded phenylalanine derivatives were chromatographed eluting with CH_2_Cl_2_/MeOH 98:2 to 95:15.

### 3.2. Nanoformulation of the Calix [8]arene-NHC-Pd Catalyst

Pd-Calix-NS were prepared by the emulsion-evaporation technique. Practically, the calix [8] arene-NHC-Pd catalyst (29.74 mg, 0.005 mmol) was dissolved into CH_2_Cl_2_ (5 mL). The organic phase was emulsified into 10 mL of 1.5% sodium cholate (*w*/*w*) aqueous solution using a vortex for 1 min and then a vibrating metallic tip at 30% amplitude for 1 min at 0 °C. The organic solvent was evaporated by magnetic stirring overnight in an open flask under a laminar flow hood and readjusted to 10 mL to provide a stock suspension of Pd-Calix-NS at 2.97 mg/mL.

### 3.3. Nanoparticles Characterization by DLS

Mean hydrodynamic diameters of the nanoassemblies and polydispersity index were measured at 25 °C by quasi-elastic light scattering with a Nano ZS (Malvern Instrument, 173 scattering angle). The NPs surface charge was investigated by ζ-potential measurement at 25 °C after dilution with 1 mM NaCl solution, applying the Smoluchowski equation. Measurements were carried out in triplicate. Colloidal stability in MilliQ water at 20 °C and 4 °C was investigated by measuring the Pd NPs mean diameter over a period of 30 days.

### 3.4. Cryogenic Transmission Electron Microscopy (CryoTEM) of Pd-Calix-NS

Morphology of the NPs of Pd-Calix-NS was observed by CryoTEM. Few drops of the nanosuspension (2.97 mg/mL) were deposited on EM grids covered with a holey carbon film (Quantifoil R2/2) previously treated with a plasma glow discharge. Observations were conducted at low temperature (−180 °C) on a JEOL 2010 FEG microscope operated at 200 kV. Images were recorded with a Gatan camera.

### 3.5. General Method for the Synthesis of Potassium Aren-2,6,7-trioxa-1-borate-bicyclo[2.2.2]octane Salts

To a solution of the given boronic acid (1.64 mmol) and 1,1,1-tris(hydroxymethyl)ethane (197 mg, 1.65 mmol) in dioxane (10 mL) was added ground KOH (92 mg, 1.64 mmol). The mixture was flushed with argon and water (29 μL, 1.65 mmol) was added. The resulting mixture was stirred at 30 °C for 3 h. Cyclohexane (5 mL) was then added and the precipitated potassium triolborate salt was filtered on a sintered glass funnel and washed twice with acetone. The solid was dried under vacuum to afford the potassium borate salts as amorphous solids.

*Potassium 4-methyl-1-phenyl-2,6,7-trioxa-1-borabicyclo-[2.2.2]octan-1-uide* (**2a**), The general method using phenyl boronic acid gave the title compound 14 in 87% yield. ^1^H NMR (300 MHz, DMSO-d_6_): δ = 7.30 ppm (d, *J* = 7.2 Hz, 2H, H-2, H-6), 6.98–6.88 (m, 3H, H-3, H-4, H-5), 3.57 (s, 6H, CH_2_OB), 0.47 (s, 3H, CH_3_) ppm; ^13^C NMR (75 MHz, DMSO-d6): δ = 132.1 (2CH, C-2, C-6), 125.5 (2CH, C-3, C-5), 124.0 (CH, C-4), 73.6 (3CH2, B(OCH_2_)_3_), 34.4 (C, CCH_3_), 16.2 (CH_3_, CCH_3_) ppm. The C-B was not observed; IR (neat, cm^−1^): 3064, 2958, 2843, 1613, 1351, 1207, 1093, 1042, 914, 878, 850, 714; MS (ESI^−^): *m*/*z* (%) = 205.1 (100) [M − K]^−^, 204.1 (20).

*Tetra-n-butylammonium 4-methyl-1-phenyl-2,6,7-trioxa-1-borabicyclo-[2.2.2]octan-1-uide* (**2e**), The general method using 1 equivalent of *n*-Bu_4_NOH as base provided the tetra-*n*-butylammonium borate salt 2e [36] in 20% yield as an off-white amorphous solid. ^1^H NMR (300 MHz, DMSO-*d*_6_): δ = 7.33 (d, *J* = 6.6 Hz, 2H, H-2,H-6), 7.02–6.85 (m, 3H, H-3,H-4, H-5), 3.58 (s, 6H, CH_2_OB), 3.20–3.10 (m, 8H, CH_2_N), 1.62–1.42 (m, 8H, CH_2_CH_2_N); 1.30 (hex, *J* = 7.3 Hz, 8H, CH_2_CH_2_CH_2_N), 0.93 (t, 7.3 Hz, 12H, CH_3_CH_2_CH_2_CH_2_N), 0.48 (s, 3H, CH_3_) ppm; ^13^C NMR (75 MHz, DMSO-*d*_6_): δ = 132.2 (2CH, C-2, C-6), 125.4 (2CH, C-3, C-5), 123.9 (CH, C-4), 73.5 (3CH_2_, B(OCH_2_)_3_), 57.5 (4CH_2_, CH_3_CH_2_CH_2_CH_2_N), 34.4 (C, CCH_3_), 23.0 (4CH_2_, CH_3_CH_2_CH_2_CH_2_N), 19.1 (4CH_2_, CH_3_CH_2_CH_2_CH_2_N), 16.2 (CH_3_, CCH_3_), 13.4 (4CH_3_, CH_3_CH_2_CH_2_CH_2_N) ppm. The C-B was not observed.

*Potassium 1-(4-methoxyphenyl)-4-methyl-2,6,7-trioxa-1-borabicyclo-[2.2.2]octan-1-uide* (**2f**), The general method using 4-methoxyphenyl boronic acid give the title compound [34] as a white powder in 69% yield; ^1^H NMR (300 MHz, DMSO-*d*_6_): δ = 7.22 (d, *J* = 8.1 Hz, 2H, H-2, H-6), 6.57 (d, *J* = 8.1 Hz, 2H, H-3, H-5), 3.63 (s, 3H, OCH_3_), 3.57 (s, 6H, CH_2_OB), 0.48 (s, 3H, CH_3_) ppm; ^13^C NMR (75 MHz, DMSO-*d*_6_): δ = 157.2 (C, C-4), 133.5 (2CH, C-2, C-6), 111.7 (2CH, C-3, C-5), 74.0 (3CH_2_, B(OCH_2_)_3_), 54.9 (CH3, OCH_3_), 34.9 (C, CCH_3_), 16.8 (CH_3_) ppm, the C-B was not observed; IR (neat, cm^−1^): 3230, 2863, 2841, 1598, 1510, 1451, 1399, 1283, 1214, 1178, 1078, 1045, 1024, 933, 854, 815,729, 697, 608; MS (ESI^−^): *m*/*z* (%) = 235.1 (100) [M-K]^−^, 234.1 (20).

*Potassium 4-methyl-1-(3-nitrophenyl)-2,6,7-trioxa-1-borabicyclo-[2.2.2] octan-1-uide* (**2h**), The general method using 3-nitrophenyl boronic acid gave the title compound [37] in 72% yield, ^1^H NMR (300 MHz, DMSO-*d*_6_): δ = 8.17 (d, *J* = 1.5 Hz, 1H, H-2), 7.83 (dd, *J* = 8.1, 1.5 Hz, 1H, H-4), 7.75 (d, 1H, *J* = 6.9 Hz, H-6), 7.29 (t, 1H, *J* = 7.6 Hz, H-5), 3.62 (s, 6H, CH_2_OB), 0.51 (s, 3H, CH_3_) ppm; ^13^C NMR (75 MHz, DMSO-*d*_6_): δ = 146.48 (C, C-3), 138.98 (CH, C-6), 126.90 (CH, C-2 or C-5), 126.30 (CH, C-2 or C-5), 119.26 (CH, C-4), 73.52 (3CH2, B(OCH_2_)_3_), 34.56 (C, CCH_3_), 15.97 (CH_3_) ppm, the C-B was not observed; IR (neat, cm^−1^): 3015, 2948, 2850, 1606, 1519, 1458, 1402, 1342, 1278, 1238, 1216, 1083, 1054, 885, 863; MS (ESI^−^): m/z (%) = 250.0 (100) [M − K]^−^, 249 (20).

*Potassium 4-methyl-1-(o-tolyl)-2,6,7-trioxa-1-borabicyclo-[2.2.2]octan-1-uide* (**2g**), The general method using *o*-tolylboronic acid gave the title compound [37] in 59% yield, ^1^H NMR (300 MHz, DMSO-*d*_6_): δ = 7.38 (d, *J* = 6.0 Hz, 1H, H-6), 6.90–6.75 (m, 3H, H-3,H-4,H-5), 3.61 (s, 6H, CH_2_OB), 2.37 (s, 3H, ArCH_3_), 0.52 (s, 3H, CH_3_) ppm; ^13^C NMR (75 MHz, DMSO-d_6_): δ = 141.6 (C, C-2), 132.4 (CH, C-6), 128.0 (CH), 124.5 (CH), 122.8 (CH), 73.0 (3CH_2_, B(OCH_2_)_3_), 34.5 (C, CCH_3_), 22.2 (CH_3_, ArCH3), 16.3 (CH_3_) ppm, the C-B was not observed; IR (neat, cm^−1^): 3158, 2855, 1396, 1210, 1178, 1066, 1035, 970, 945, 928, 891, 829, 750, 689; MS (ESI^−^): *m*/*z* (%) = 219.1 (100) [M-K]^−^, 218 (20).

*Potassium 1-(furan-2-yl)-4-methyl-2,6,7-trioxa-1-borabicyclo-[2.2.2]octan-1-uide* (**2i**), The general method using 2-furylboronic acid gave the title compound [38] in yield 67%; ^1^H NMR (300 MHz, DMSO-d_6_): δ = 7.31 (s, 1H, H-5), 6.10 (m, 1H, H-3), 5.94 (d, *J* = 3.0 Hz, 1H, H-4), 3.54 (s, 6H, CH_2_OB), 0.48 (s, 3H, CH_3_) ppm; ^13^C NMR (75 MHz, DMSO-d_6_): δ = 140.6 (CH, C-5), 110.3 (CH, C-3 or C-4), 108.4 (CH, C-3 or C-4), 72.9 (3CH_2_, B(OCH_2_)_3_), 34.4 (C, CCH_3_), 16.1 (CH_3_) ppm, the C-B was not observed; MS (APCI^−^): m/z (%) = 195.1 (100) [M^−^], 194.1 (20).

*Potassium 4-methyl-1-(thiophen-3-yl)-2,6,7-trioxa-1-borabicyclo-[2.2.2]octan-1-uide* (**2j**), The general method using 3-thienylboronic acid gave the title compound [39] in yield 65%; ^1^H NMR (300 MHz, DMSO-d_6_): δ = 7.01 (dd, J = 4.5 Hz, *J* = 2.7 Hz, 1H, H-5), 6.93 (d, *J* = 4.5 Hz 1H, H-4), 6.85 (d, *J* = 1.8 Hz, 1H, H-2), 3.54 (s, 6H, CH_2_OB), 0.46 (s, 3H, CH_3_) ppm; ^13^C NMR (75 MHz, DMSO-d_6_): δ = 132.9 (CH, C-4), 123.7 (CH, C-2), 120.9 (CH, C-5), 73.6 (3CH_2_, B(OCH_2_)_3_), 34.4 (C, CCH_3_), 16.2 (CH_3_) ppm, the C-B was not observed; IR (neat, cm^−1^): 2948, 2847, 1630, 1468, 1421, 1400, 1351, 1215, 1172, 1071, 1000, 926, 869, 839, 774, 599, 622; MS (ESI^−^): m/z (%) = 211.2 (5) [M − H]^−^, 195.1 (100).

### 3.6. General Method for the Small-Scale Suzuki-Miyaura Coupling with Pd-Calix-NS

In an Eppendorf tube, the arylhalide (0.008 mmol, 1 equiv.) and the boronic acid/cyclic-triolborate salt (0.024 mmol, 3 equiv.) were suspended in phosphate buffer pH 8.0–6.0 (200 mM, 50 μL) and MilliQ Water (370 µL). The suspension of Pd-Calix-NS was added (3.2·10^−6^ mmol; 0.00004 equiv.; 80 µL) and the reaction was mixed and shaken on a thermostated shaker (Biosan TS-100) at 800 rpm at 37 °C. After 3 h, the reaction was frozen in liquid nitrogen and the solution lyophilized. The crude product was taken up in CD_3_OD and analyzed by ^1^H-NMR.

### 3.7. General Method for the Preparative Suzuki-Miyaura Coupling with Pd-Calix-NS

The arylhalide (0.25 mmol, 1 equiv.) and the boronic acid (0.75 mmol, 3 equiv.) were suspended in phosphate buffer pH 8.0 (200 mM, 5 mL) and water (The stock solution of Pd-Calix-NS (0.025 mmol Pd, 0.01 equiv.) was added and the reaction was stirred at 37 °C. After 3/16 h, the reaction was frozen in liquid nitrogen and the solution was lyophilized. The crude product was purified by flash chromatography on silica gels to afford the expected cross-coupling product.

*4-Phenylbenzoic Acid* (**3e**), The general method using 4-iodobenzoic acid and potassium phenyltriolborate gave 4-phenylbenzoic acid [40] (Yield = 72%); ^1^H NMR (300 MHz, CDCl_3_): δ = 8.19 (d, *J* = 8.2 Hz, 2H, H-2**′**, H-6**′**), 7.71 (d, *J* = 8.2 Hz, 2H, H-3**′**, H-5**′**), 7.64 (d, *J* = 7.8 Hz, 2H, H-2”, H-6”), 7.55–7.38 (m, 3H, H3”, H-4”, H-5”) ppm; ^13^C NMR (75 MHz, DMSO-d_6_): δ = 167.2 (C, CO_2_H), 144.3 (C), 139.1 (C), 130.0 (2CH), 129.7 (C), 129.1 (2CH), 128.3 (CH), 127.0 (2CH), 126.8 (2CH), ppm; MS (ESI^−^) m/z = 197.1 (100) [M − H]^−^.

*4-Phenylphenol* (**3f**), The general method using 4-iodophenol and potassium phenyltriolborate gave 4-phenylphenol [41] (Yield = 90%); ^1^H NMR (300 MHz, CD_3_OD): δ = 10.38 (1H, br s, OH), 8.37 (d, *J* = 8.1 Hz, 2H, H-2′, H-6′), 8.28 (d, *J* = 8.7 Hz, 2H, H-3, H-5), 8.21 (t, J = 7.5 Hz, 2H, H-3′, H-5′), 8.07 (td, *J* = 7.2, 0.9 Hz, 1H, H-4′), 7.66 (d, *J* = 8.4 Hz, 2H, H-2, H-6) ppm; ^13^C NMR (75 MHz, CD_3_OD): δ = 160.6 (C, C-1), 149.7 (C, C-1′), 140.5 (C, C-4), 138.3 (2CH, C-3, C-5), 137.3 (2CH, C-3′, C-5′), 135.9 (CH, C-4′), 135.5 (2CH, C-2′, C-6′), 125.3 (2CH, C-2, C-6) ppm; MS (ESI^−^) m/z (%) = 169.1 (100) [M − H]^−^.

*2-Phenylbenzoic Acid* (**3h**), The general method using 2-iodobenzoic acid and potassium phenyltriolborate gave 2-phenylbenzoic acid [42] (Yield = 66%) ^1^H NMR (300 MHz, DMSO-d_6_): δ = 12.70 (s, 1H, OH), 7.72 (d, *J* = 7.5 Hz, 1H, H-6, 1H), 7.57 (t, *J* = 7.5 Hz, 1H, H-4, 1H), 7.45 (t, *J* = 7.3 Hz, 1H, H-5, 1H), 7.45–7.30 (m, 6H) ppm; ^13^C NMR (75 MHz, CDCl_3_): δ = 173.6 (C, CO_2_H), 143.5 (C, C-2), 141.1 (C, C-1**′**), 132.2 (CH), 131.3 (CH), 130.8 (CH), 129.4 (C, C-1), 128.6 (2CH), 128.2 (2CH), 127.5 (CH), 127.3 (CH) ppm.

*4-Phenylaniline* (**3i**), The general method using 4-iodooaniline and potassium phenyltriolborate gave 4-phenylaniline [43] (Yield = 70%) ^1^H NMR (300 MHz, DMSO-d_6_): δ = 7.52 (d, *J* = 8.2 Hz, 2H, H-2, H-5, 2H), 7.42–7.32 (m, 4H, H-2**′**, H-3**′**, H-5**′**, H-6**′**), 7.20 (t, J = 7.3 Hz, 1H, H-4**′**), 6.67 (d, *J* = 8.2 Hz, 2H, H-2, H-6), 5.19 (br s, 2H, NH_2_) ppm; ^13^C NMR (75 MHz, DMSO-d_6_): δ = 148.3 (C), 140.7(C), 128.7 (2CH), 127.5 (C), 127.1 (2CH), 125.6 (CH), 125.3 (2CH), 114.3 (2CH) ppm; MS (ESI^+^) m/z (%) = 170.1 (100) [M + H]^+^.

*2-Phenylaniline* (**3j**), The general method using 2-iodooaniline and potassium phenyltriolborate gave 2-phenylaniline [44] (Yield = 32%) ^1^H NMR (300 MHz, CD_3_OD): δ = 7.50–7.25(m, 5H), 7.09 (dt, *J* = 8.1 Hz, 1.5 Hz, 1H, H-5), 7.02 (d, *J* = 7.8 Hz, 1H, H-3), 6.81 (d, *J* = 7.8 Hz, 1H, H-6), 6.75 (t, *J* = 7.3 Hz, 1H, H-4) ppm; ^13^C NMR (75 MHz, CD_3_OD): δ = 144.85 (C, C-2), 139.7 (C, C-1**′**), 130.0 (CH), 128.7 (2CH, C-3**′**, C-5**′**), 128.6 (2CH, C-2**′**, C-6**′**), 128.2 (CH), 126.7 (CH), 125.9 (C, C-1), 116.8 (CH, C-5), 115.3 (CH, C-3) ppm; MS (ESI^+^) *m*/*z* (%) = 170.1 (100) [M + H]^+^.

*N-boc-4-phenyl-l-phenylalanine* (**3a**), The general method using *N*-Boc-4-iodo-l-phenylalanine and potassium phenyltriolborate (2a) gave the title compound [45] in 87% yield: ^1^H NMR (300 MHz, CD_3_OD) δ 7.62–7.48 (m, 4H), 7.40 (t, J = 7.5 Hz, 2H), 7.35–7.27 (m, 3H), 4.46–4.30 (m, 1H, H-2), 3.20 (dd, J = 13.8, 5.1 Hz, 1H, H-3), 2.95 (dd, J = 13.8, 8.7 Hz, 1H, H-3), 1.40 (s, 9H, NCO2C(CH_3_)_3_) ppm; ^13^C NMR (75 MHz, CD_3_OD): δ = 175.4 (C, CO_2_H), 157.8 (C, CO_2_*t*-Bu), 142.3 (C), 141.0 (C), 137.8 (C), 130.8 (2CH), 129.8 (2CH), 128.2 (CH), 128.0 (2CH), 127.9 (2CH), 80.5 (C, NCO_2_C(CH_3_)_3_)), 56.3 (CH, C-2), 38.4 (CH2, C-3), 28.7 (3CH3, NCO_2_C(CH_3_)_3_) ppm; MS (ESI-) *m*/*z* (%): 340.17 (100) [M − H]^−^, 266.2 (80) [M-t-BuOH-H]^−^.

*N-boc-3-phenyltyrosine* (**3d**), The general method using *N*-Boc-4-iodo-l-tyrosine and potassium phenyltriolborate (2a) gave the title compound [46] in 68% yield; ^1^H NMR (300 MHz, CD_3_OD): δ = 7.55 (d, *J* = 7.3 Hz, 2H, H-2”, H-6”), 7.36 (t, *J* = 7.3 Hz, 2H, H-3”, H-5”), 7.26 (t, *J* = 7.3 Hz, 1H, H-4**”**), 7.12 (s, 1H, H-2**′**), 7.02 (d, *J* = 8.2 Hz, 1H, H-6**′**), 6.82 (d, *J* = 8.2 Hz, 1H, H-5**′**), 4.45–4.25 (m, 1H, H-3), 3.10 (dd, *J* = 13.1, 1.8 Hz, 1H, H-3), 2.89 (dd, *J* = 13.1, 7.7 Hz, 1H, H-3), 1.37 (s, 9H, NCO2C(CH_3_)_3_) ppm; ^13^C NMR (75 MHz, CD_3_OD): δ = 175.4 (C, CO_2_H), 157.8 (C, CO_2_*t*-Bu), 154.1 (C, C-4**′**), 140.2 (C, C-1**′**), 132.7 (C), 130.4 (2CH), 130.2 (CH), 129.6 (C), 129.5 (C), 128.9 (2CH), 127.6 (CH), 117.0 (CH), 80.5 (C, NCO_2_C(CH_3_)), 56.4 (CH, C-2), 37.9 (CH2, C-3), 28.7 (3CH3, NCO_2_C(CH_3_)_3_) ppm; MS m/z (%) (ESI^−^): 356.2 (100) [M − H]^−^, 282.2 (65) [M-t-BuOH-H]^−^.

*N-Boc-4-(4-methoxyphenyl)-l-phenylalanine* (**3af**), The general method using *N*-Boc-4-iodo-l-phenylalanine and potassium 4-methoxy-1-phenyl-2,6,7-trioxa-1-borabicyclo[2.2.2]octan-1-uide (2f) gave the title compound [47] in 98% yield; ^1^H NMR (300 MHz, CD_3_OD): δ = 7.49 (t, *J* = 8.4 Hz, 4H, H-2**′**, H-6**′**, H-2”, H-6”), 7.27 (d, *J* = 7.8 Hz, 2H, H-2**′**, H-6**′**), 6.97 (d, *J* = 8.7 Hz, 2H, H-3”, H-5”), 4.45–4.30 (m, 1H, H-2), 3.82 (s, 3H, OMe), 3.18 (dd, *J* = 13.8, 4.8 Hz, 1H, H-3), 2.95 (dd, *J* = 13.8, 9.3 Hz, 1H, H-3), 1.38 (s, 9H, NCO2C(CH_3_)_3_) ppm; ^13^C NMR (75 MHz, CD_3_OD): δ = 175.4 (C, CO2H), 160.7 (C-4”), 157.7 (C, CO2t-Bu), 140.6 (C, C-1**′**), 137.0 (C), 134.7 (C), 130.8 (2CH), 128.8 (2CH), 127.5 (2CH), 115.2 (2CH, C-3”, C-5”), 80.5 (C, NCO_2_C(CH_3_)), 56.4 (CH, C-2), 55.7 (CH_3_, OCH_3_), 38.4 (CH_2_, C-3), 28.7 (3CH3, NCO_2_C(CH_3_)_3_) ppm.

*N-boc-4-(2-methylphenyl)-l-phenylalanine* (**3ag**), The general method using *N*-Boc-4-iodo-l-phenylalanine and potassium 2-methyl-1-phenyl-2,6,7-trioxa-1-borabicyclo[2.2.2]octan-1-uide (2g) gave the title compound [46] in 79% yield after 16 h reaction time. ^1^H NMR (300 MHz, CD_3_OD): δ = 7.30 (d, *J* = 8.7 Hz, 2H), 7.28–7.10 (m, 6H), 4.45–4.30 (m, 1H, H-2), 3.21 (dd, *J* = 13.8, 4.5 Hz, 1H, H-3), 2.95 (dd, *J* = 13.8, 9.3 Hz, 1H, H-3), 2.21 (s, 3H, ArCH_3_), 1.39 (s, 9H, NCO_2_C(CH_3_)_3_) ppm; ^13^C NMR (75 MHz, CD_3_OD): δ = 175.4 (C, CO_2_H), 157.8 (C, CO_2_*t*-Bu), 143.0 (C, C-1**′**), 141.8 (C), 137.2 (C), 136.2 (C), 131.2 (CH), 130.6 (CH), 130.1 (4CH), 128.2 (CH), 126.7 (CH), 80.5 (C, NCO_2_C(CH_3_)), 56.3 (CH, C-2), 38.5 (CH_2_, C-3), 28.7 (3CH_3_, NCO_2_C(CH_3_)_3_), 20.6 (CH3, ArCH_3_) ppm; MS m/z (%) (ESI^−^): 354.2 (100) [M − H]^−^, 280.2 (85) [M-t-BuOH-H]^−^.

*N-boc-4-(3-nitrophenyl) phenylalanine* (**3ah**), The general method using *N*-Boc-4-iodo-l-phenylalanine and potassium 3-nitro-1-phenyl-2,6,7-trioxa-1-borabicyclo[2.2.2]octan-1-uide (2h) gave the title compound [47] in 72% yield after 16 h reaction time; 1H NMR (300 MHz, CD_3_OD): δ = 8.45 (s, 1H, H-2”), 8.19 (d, J = 8.0 Hz, 1H, H-4”), 8.02 (d, *J* = 7.9 Hz, 1H, H-6”), 7.68 (t, *J* = 7.9 Hz, 1H, H-5”), 7.63 (d, *J* = 8.1 Hz, 2H, H-3**′**, H-5**′**), 7.39 (d, *J* = 8.1 Hz, 2H, H-2**′**, H-6**′**), 4.45–4.30 (m, 1H, H-2), 3.24 (dd, *J* = 13.8, 4.8 Hz, 1H, H-3), 2.98 (dd, *J* = 13.8, 8.4 Hz, 1H, H-3), 1.39 (s, 9H, NCO_2_C(CH_3_)_3_) ppm; ^13^C NMR (75 MHz, CD_3_OD): δ = 175.2 (C, CO_2_H), 157.8 (C, CO_2_*t*-Bu), 150.2 (C, C-3”), 144.0 (C), 139.4 (C), 138.4 (C), 134.0 (CH), 131.3 (2CH), 131.2 (CH), 128.4 (2CH), 122.9 (CH), 122.4 (CH), 80.6 (C, NCO_2_C(CH_3_)), 56.2 (CH, C-2), 38.4 (CH_2_, C-3), 28.7 (3CH_3_, NCO_2_C(CH_3_)_3_), MS m/z (%) (ESI^−^): 385.2 (100) [M − H]^−^, 311.2 (65) [M-*t*-BuOH-H]^−^.

*N-boc-4-(2-furanyl) phenylalanine* (**3ai**), The general method using *N*-Boc-4-iodo-l-phenylalanine and potassium 4-methyl-1-(furan-2-yl)-2,6,7-trioxa-1-borabicyclo[2.2.2]octan-1-uide (2i) gave the title compound in 87% yield ^1^H NMR (300 MHz, CD_3_OD): δ = 7.60 (d, *J* = 8.0 Hz, 2H, H-3**′**, H-5**′**), 7.51 (s, 1H, H-4”), 7.25 (d, *J* = 8.0 Hz, 2H, H-2**′**, H-6**′**), 6.70 (d, *J* = 2.8 Hz, 1H, H-3”), 6.50 (br s 1H, H-4”), 4.45–4.25 (m, 1H, H-2), 3.16 (dd, *J* = 13.8, 4.5, Hz, 1H, H-3), 2.92 (dd, *J* = 13.8, 9.3 Hz, 1H, H-3), 1.39 (s, 9H, NCO_2_C(CH_3_)_3_); ^13^C NMR (75 MHz, CD_3_OD): δ = 175.3 (C, CO_2_H), 157.7 (C, CO_2_*t*-Bu), 155.2 (C, C-2”), 143.2 (CH), 137.8 (C), 130.7 (2CH, C-2**′**, C-6**′**), 129.8 (C), 124.7 (2CH, C-3**′**, C-5**′**), 112.6 (CH, C-3”), 105.7 (CH, C-4”), 80.5 (C, NCO_2_C(CH_3_)), 56.2 (CH, C-2), 38.5 (CH2, C-3), 28.6 (3CH_3_, NHCO_2_C(CH_3_)_3_); MS m/z (%) (ESI^−^): 330.2 (100) [M − H]^−^, 256.2 (85) [M-tBuOH-H]^−^.

*N-boc-4-(4-thienyl) phenylalanine* (**3aj**), The general method using *N*-Boc-4-iodo-l-phenylalanine and potassium 4-methyl-1-(thiophen-3-yl)-2,6,7-trioxa-1-borabicyclo[2.2.2]octan-1-uide (2j) gave the title compound [48] in 63% yield ^1^H NMR (300 MHz, CD_3_OD): δ = 7.58–7.49 (m, 3H, H-2”, H-3**′**, H-5**′**), 7.45–7.38 (m, 2H, H-4”, H-5”), 7.25 (d, *J* = 7.8 Hz, 2H, H-2**′**, H-6**′**), 4.35–4.20 (m, 1H, H-2), 3.18 (dd, *J* = 13.5, 4.5, Hz, 1H, H-3), 2.94 (dd, *J* = 13.5, 7.2 Hz, 1H, H-3), 1.38 (s, 9H, NCO_2_C(CH_3_)_3_); ^13^C NMR (75 MHz, CD_3_OD): δ = 175.4 (C, CO_2_H), 157.8 (C, CO_2_*t*-Bu), 143.3 (C, C-3”), 137.5 (C), 135.7 (C), 130.8 (2CH, C-2**′**, C-6**′**), 127.9 (2CH, C-3**′**, C-5**′**), 127.2 (CH, C-3” or C-4”), 127.1 (CH, C-3” or C-4”), 120.9 (CH, C-2”), 80.5 (C, NCO_2_C(CH_3_)), 56.3 (CH, C-2), 38.4 (CH2, C-3), 28.7 (3CH3, NHCO_2_C(CH_3_)_3_); MS (ESI^−^): *m*/*z* (%) 346.2 (100) [M − H]^−^, 272.1 (50) [M-*t*-BuOH-H]^−^.

## 4. Conclusions

To conclude, we have shown that the nanoformulation of benzyloxycalix [8] arene supported NHC-palladium complexes gave highly stable nanosuspension endowed with high catalytic activity for the Suzuki–Miyaura couplings in pure water. In combination with cyclic triol boronates as nucleophilic partner, this nanoformulation was successfully engaged in coupling reactions with water-soluble substrates bearing a wide range of functional groups using a low catalyst loading.

The high catalytic activity of the Pd-Calix-NS can be attributed to the specific characteristics of the nanostructure exalting the high catalytic power the NHC-Pd structure previously observed in organic solvents [27,49]. The exact origin of the increase reactivity of the nanoparticulate formulation remain to be addressed, nevertheless it may be hypothesis that the self-assembling of the calix molecules driven by hydrophobic interaction would give NPs embedding the lipophilic crown of benzyl groups into the core of the particles, while the more polar palladium NHC appendages would be disposed on the surface. Such an arrangement would favorably influence the oxidative addition step with water soluble aryl halides and hence increase the rate of the overall process.

## Figures and Tables

**Table 1:** Size, polydispersity index (PDI) and ζ-Potential of the Pd-Calix-NPs as measured by dynamic light scattering according to the method of preparation.

Method of Formulation	Size (nm)	PDI	ζ-Potential (mV)
Nanoprecipitation	116	0.18	−3.14
Emulsion evaporation	112	0.21	−16.8

**Table 2:**
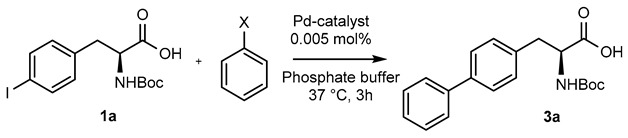
Influence of the organoboron derivative on the cross-coupling reaction of *N*-Boc-4-iodo-l-phenylalanine.

Entry	Pd Catalyst	X	Conv (%) ^a^
**1**	Pd-Calix-NS	B(OH)_2_	40
**2**	Pd-Calix-NS	BF_3_-K^+^	10
**3**	Pd-Calix-NS	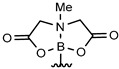	0
**4**	Pd-Calix-NS	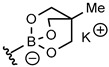	98
**5**	Bulk Pd-Calix	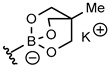	<5
**6**	Pd-PLGA-PEG NPs	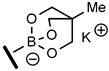	97^b^
**7**	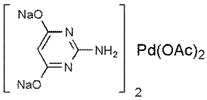	B(OH)_2_	95^c^

^a^*N*-Boc-4-iodo-l-phenylalanine (1 equiv.), boronic acid derivatives (3 equiv.), Pd-Calix-NS (0.005 mol%), phosphate buffer (20 mM, pH = 8), 37 °C for 3 h. Conversion determined by ^1^H NMR analysis of the C-3 methylene signal. ^b^ 0.01% Pd, according to reference [30]. ^c^ 1 mol% Pd, according to reference [18].

**Table 3:**
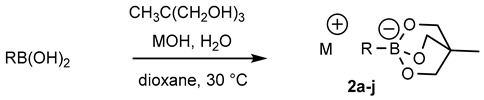
Synthesis of cyclic triolborates from boronic acids.

Compound	R	M	Time (h)	Yield (%)
**2a**	Ph	K	3	80
**2b**	Ph	Li	3	46
**2c**	Ph	Na	3	87
**2d**	Ph	Cs	3	67
**2e**	Ph	*n*-Bu_4_N	3	20
**2f**	*p*-MeOPh	K	3	69
**2g**	*o*-Tol	K	16	59^a^
**2h**	*m*-NO_2_Ph	K	16	72
**2i**	2-furanyl	K	3	67
**2j**	3-thiophenyl	K	3	65

^a^ The reaction was conducted at 60 °C.

**Table 4:**
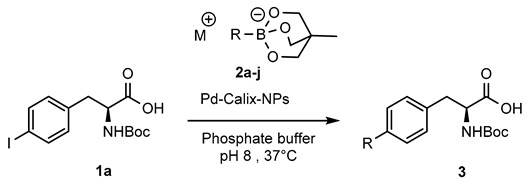
Reactivity of aryl and heteroaryl cyclic triolborates with *N*-Boc-4-iodophenylalanine.

Entry	Cyclic borate	R	Metal	pH	Product	Yield(%) ^a^
**1**	**2a**	Ph	K	8.0	**3a**	98
**2**	**2a**	Ph	K	7.0	**3a**	67
**3**	**2a**	Ph	K	6.0	**3a**	56
**4**	**2b**	Ph	Li	8.0	**3a**	30
**5**	**2c**	Ph	Na	8.0	**3a**	90
**6**	**2d**	Ph	Cs	8.0	**3a**	47
**7**	**2e**	Ph	TBA	8.0	**3a**	22
**8**	**2f**	*p*-MeOPh	K	8.0	**3af**	98
**9**	**2g**	*o*-Tol	K	8.0	**3ag**	79^b^
**10**	**2h**	*m*-NO_2_Ph	K	8.0	**3ah**	72^b^
**11**	**2i**	2-furanyl	K	8.0	**3ai**	87
**12**	**2j**	3-thiophenyl	K	8.0	**3aj**	63

^a^ The reaction was conducted at 60 °C.

## Supplementary Materials

Copies of ^1^H NMR and ^13^C NMR spectra of the compound are available in the online supplementary materials.
