# Supplementary material for: A Self-Assembling NHC-Pd-Loaded Calixarene as a Potent Catalyst for the Suzuki-Miyaura Cross-Coupling Reaction in Water
Source: Molecules. 2020 Mar 24;25(6):1459. doi: 10.3390/molecules25061459 (PMC7146153; doi:10.3390/molecules25061459)
Supplement: Supplementary file 1 [file molecules-25-01459-s001.pdf]

A Self-Assembling NHC-Palladium-Loaded Calixarene as a Potent Catalyst for the Suzuki-Miyaura Cross-coupling Reaction in Water

Arnaud Peramo,[1] *Ibrahim Abdellah,*[2] *Shannon Pecnard,*[1] *Julie Mougin*[1], *Cyril Martini,*[2,3] *Patrick Couvreur,*[1] *Vincent Huc,*[2,]* and *Didier Desmaële*[1]*

[1] *Institut Galien Paris Sud, CNRS UMR8612, Université Paris Saclay, 5 rue Jean-Baptiste Clément 92290 Châtenay-Malabry, France*

[2] *Institut de Chimie Moléculaire et des Matériaux d'Orsay, CNRS UMR8182, Université Paris Saclay, Bâtiment 420, 91405 Orsay, France.*

[3] *NOVECAL, 86 rue de Paris, 91400 Orsay, France, www.novecal.com*

Email : vincent.huc@u-psud.fr and didier.desmaele@u-psud.fr

^{1}H and ^{13}C NMR Spectra of the synthesized compounds

Compound **2a** page 2
Compound **2e** page 3
Compound **2f** page 4
Compound **2g** page 5
Compound **2h** page 6
Compound **2i** page 7
Compound **2j** page 8
Compound **3a** page 9
Compound **3d** page 10
Compound **3e** page 11
Compound **3f** page 12
Compound **3h** page 13
Compound **3i** page 14
Compound **3j** page 15
Compound **3af** page 16
Compound **3ag** page 17

Compound **3ah** page 18
Compound **3ai** page 19
Compound **3aj** page 20

^{1}H NMR **2a**

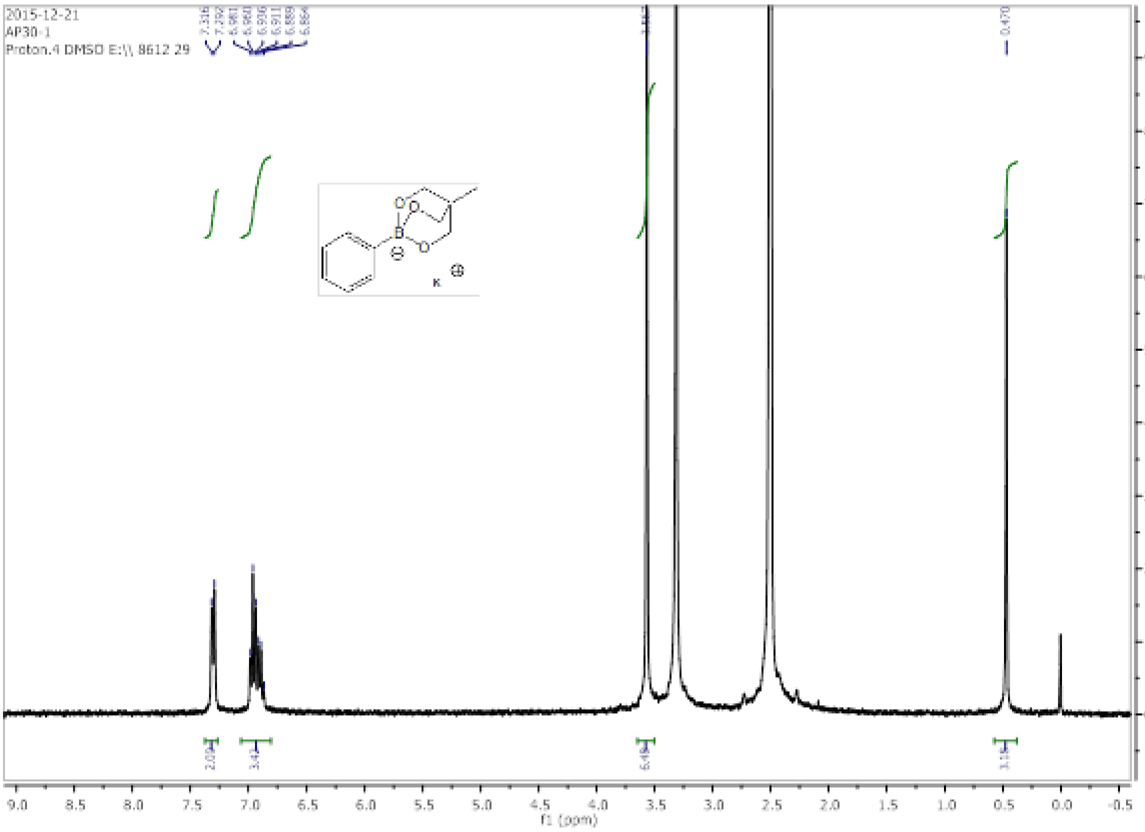

^{13}C NMR of **2a**

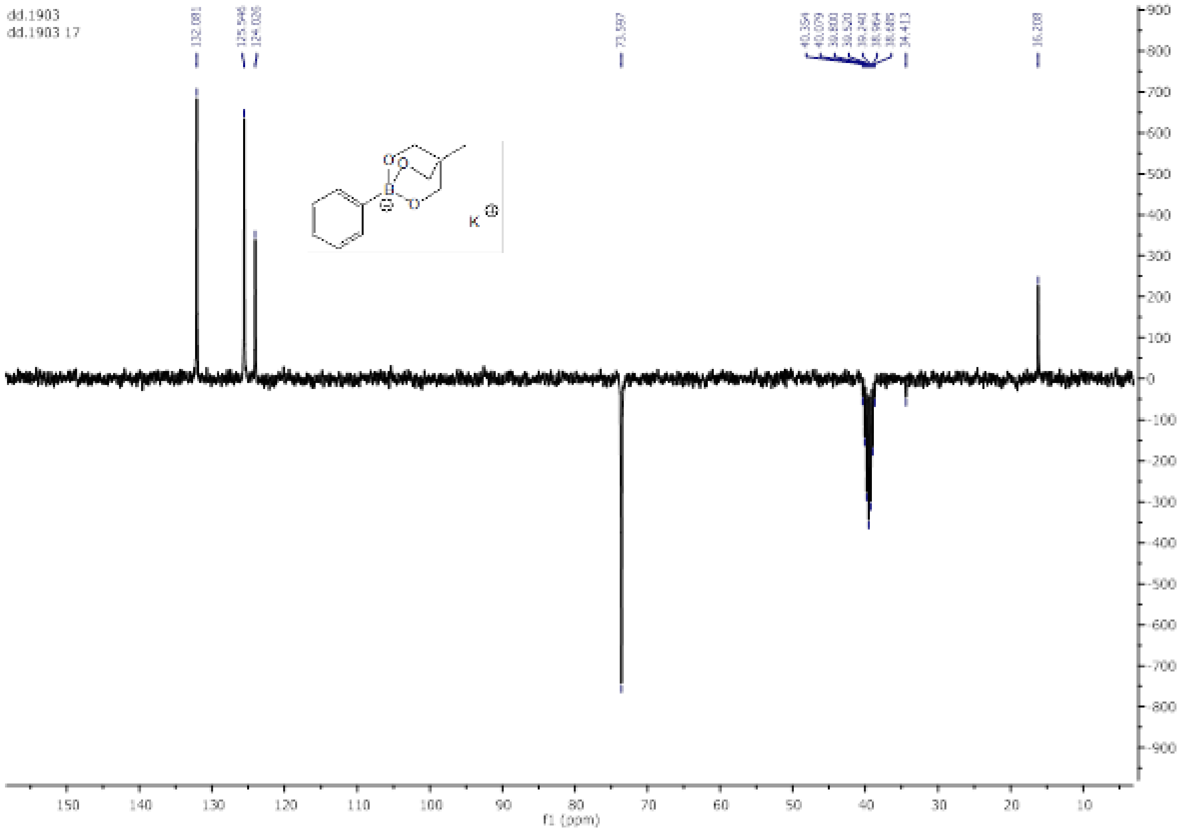

1H NMR **2e**

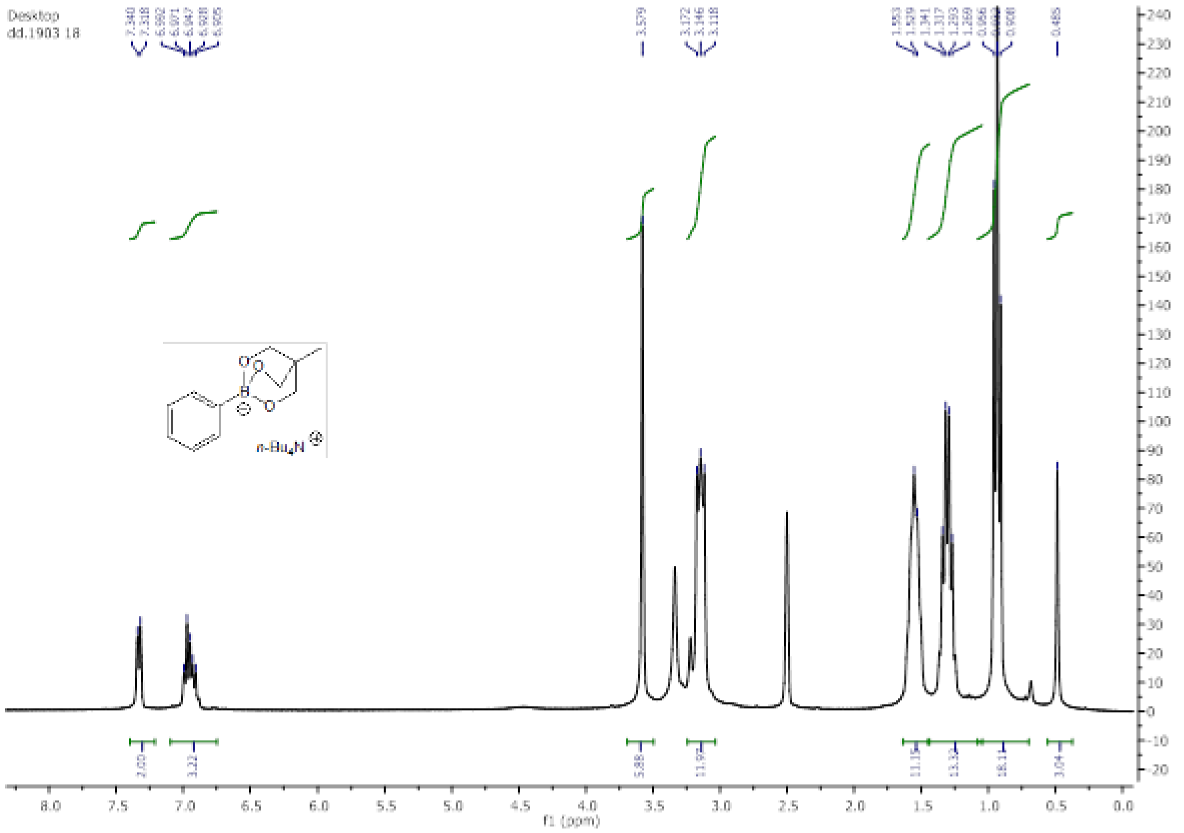

^{13}C NMR **2e**

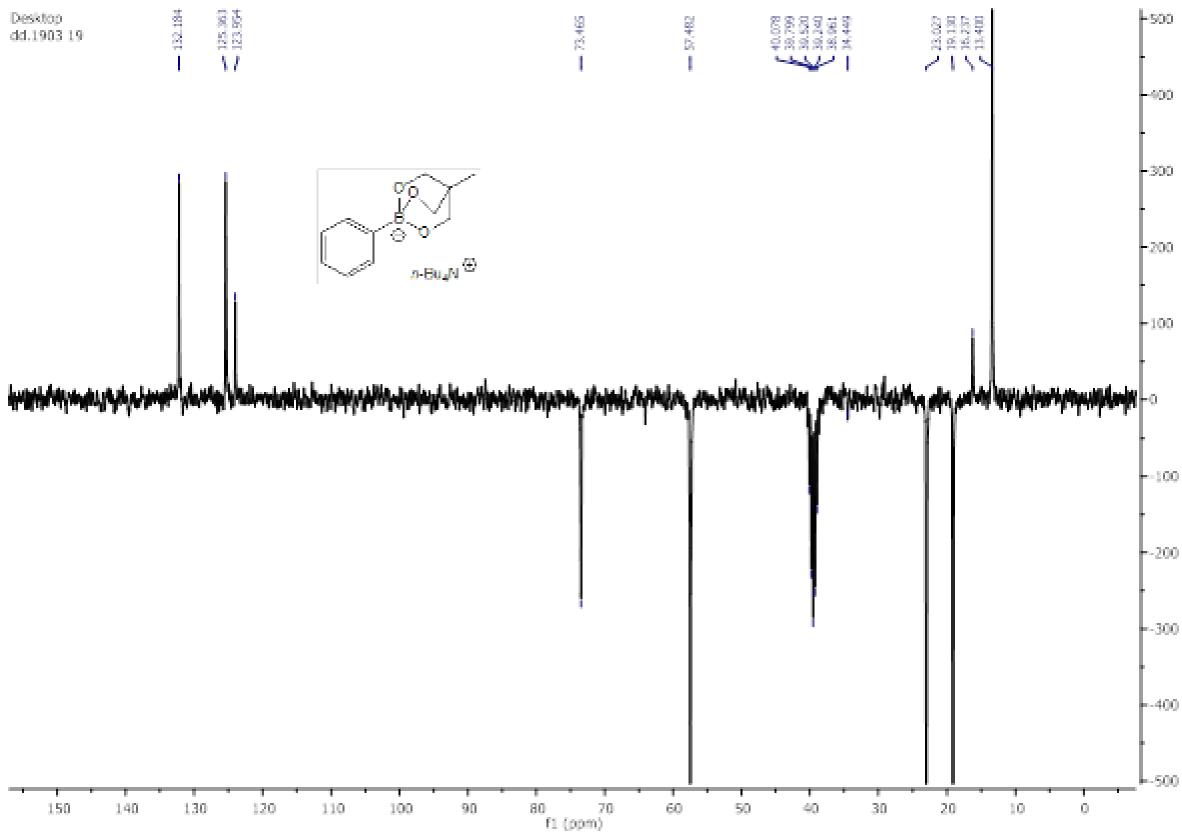

^{1}H NMR **2f**

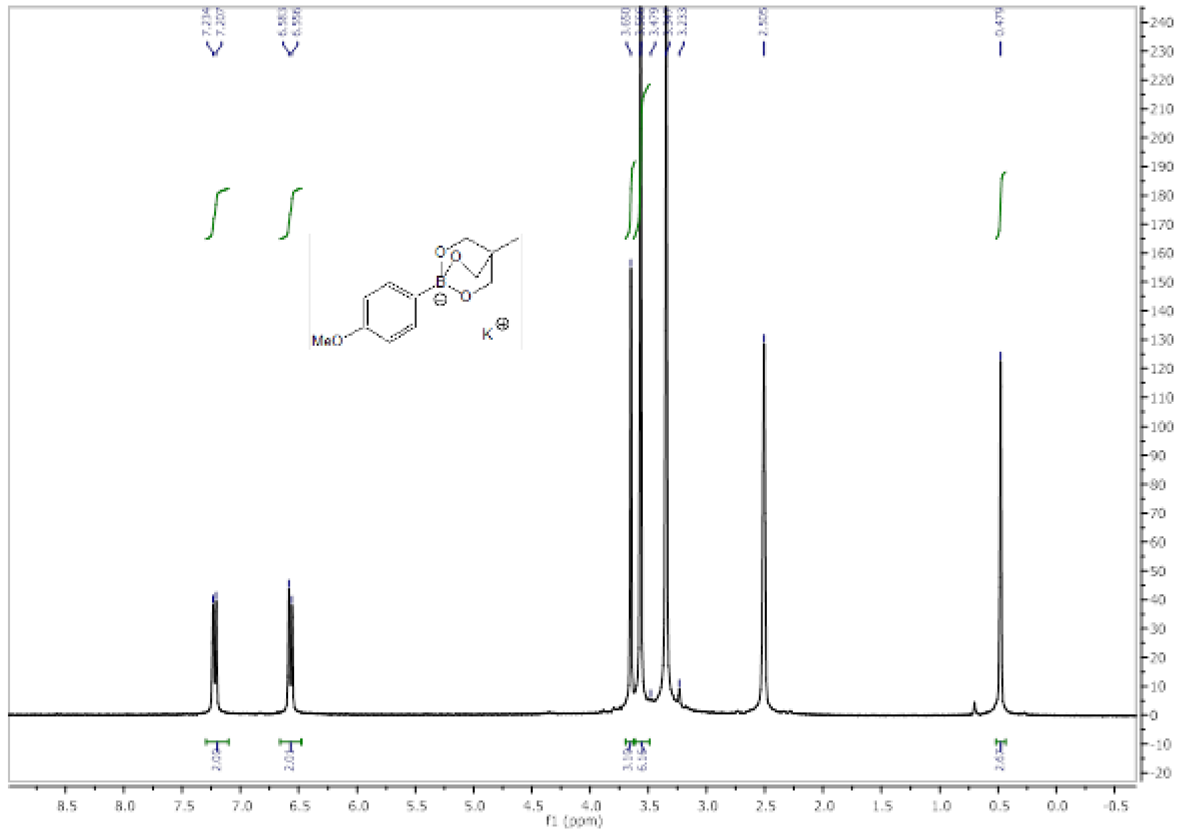

^{13}C NMR **2f**

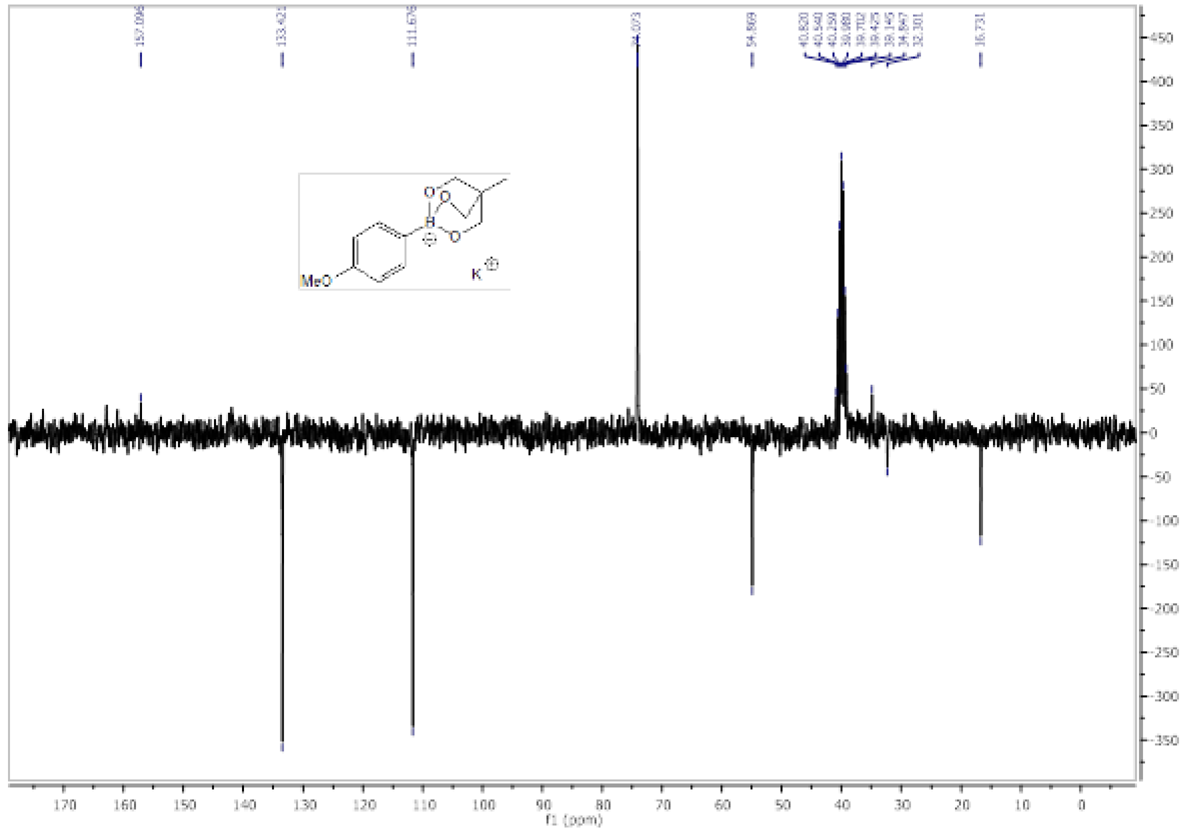

^{1}H NMR **2g**

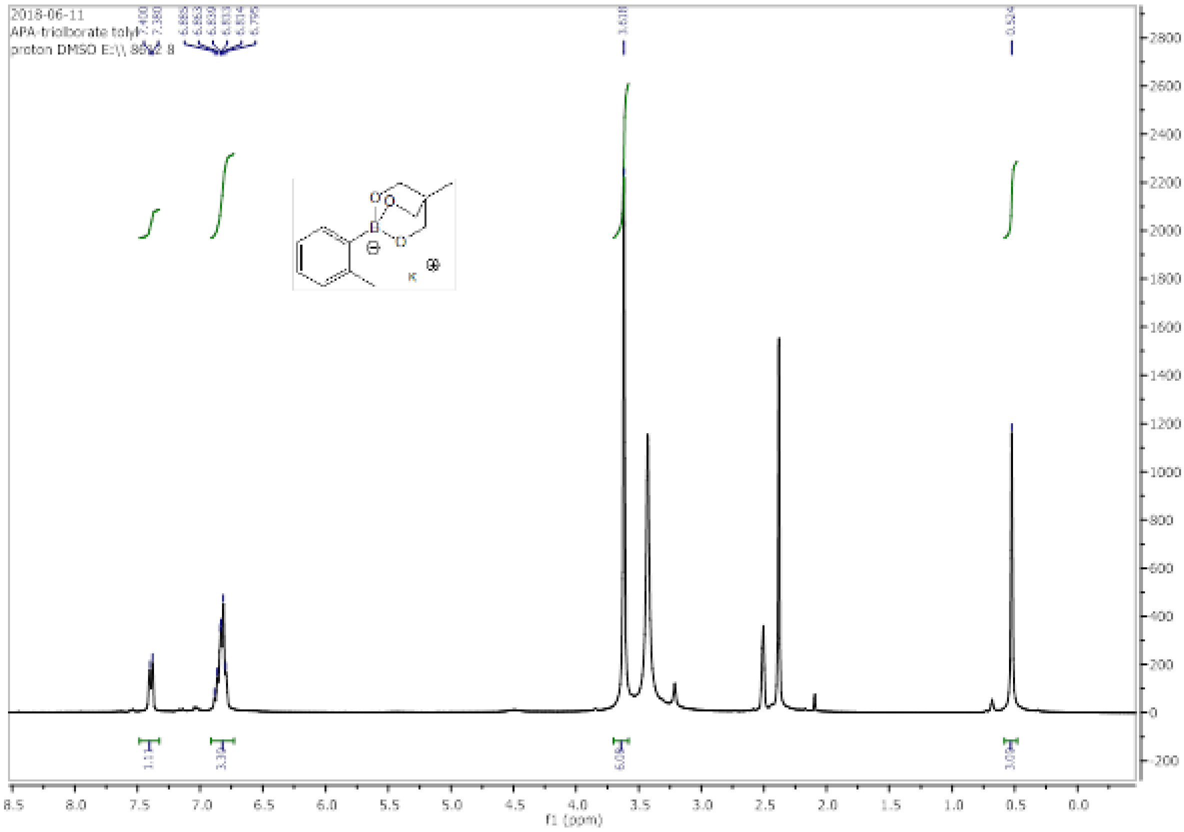

^{13}C NMR **2g**

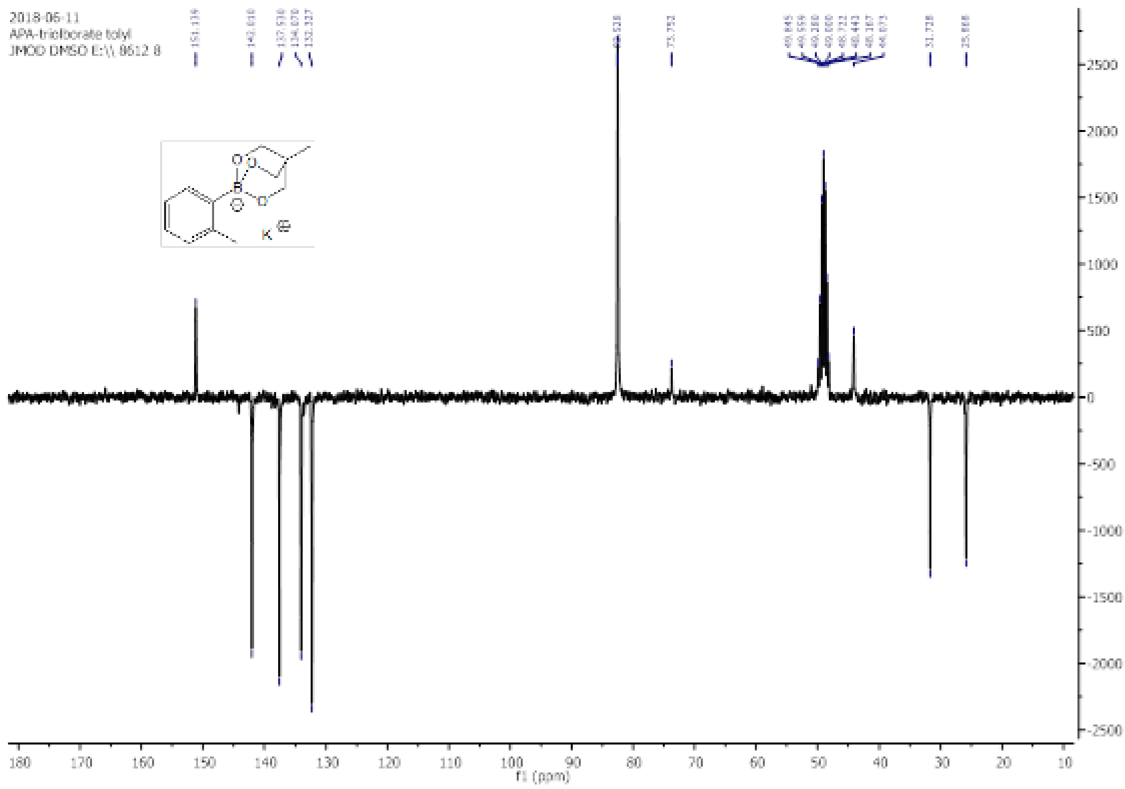

^{1}H NMR **2h**

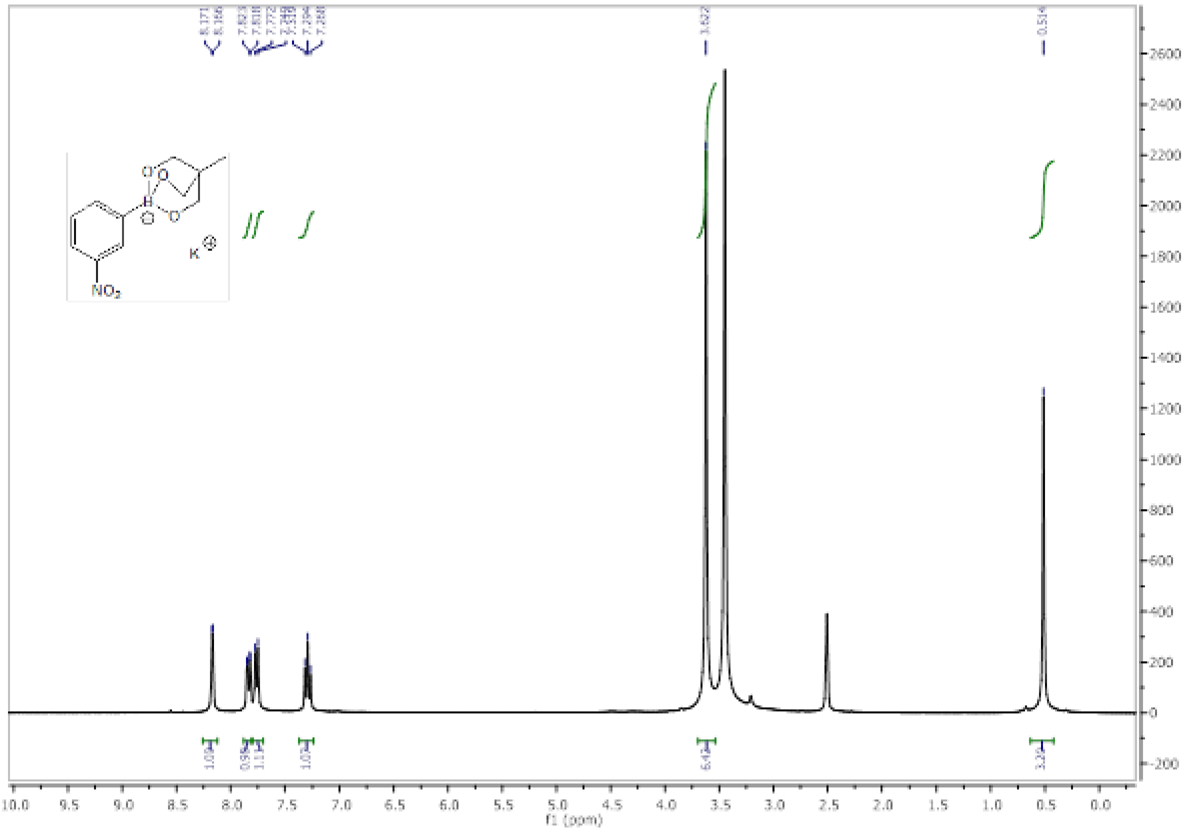

^{13}C NMR **2h**

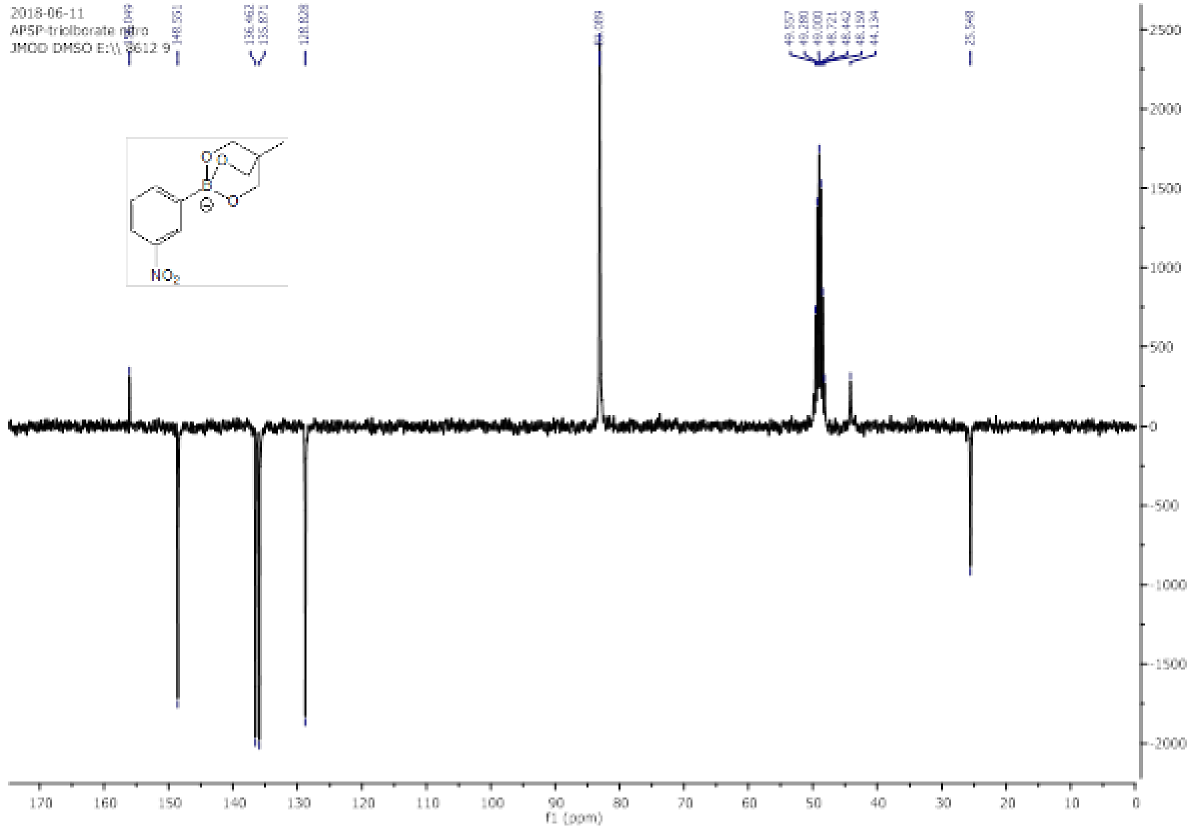

^{1}H NMR of **2i**

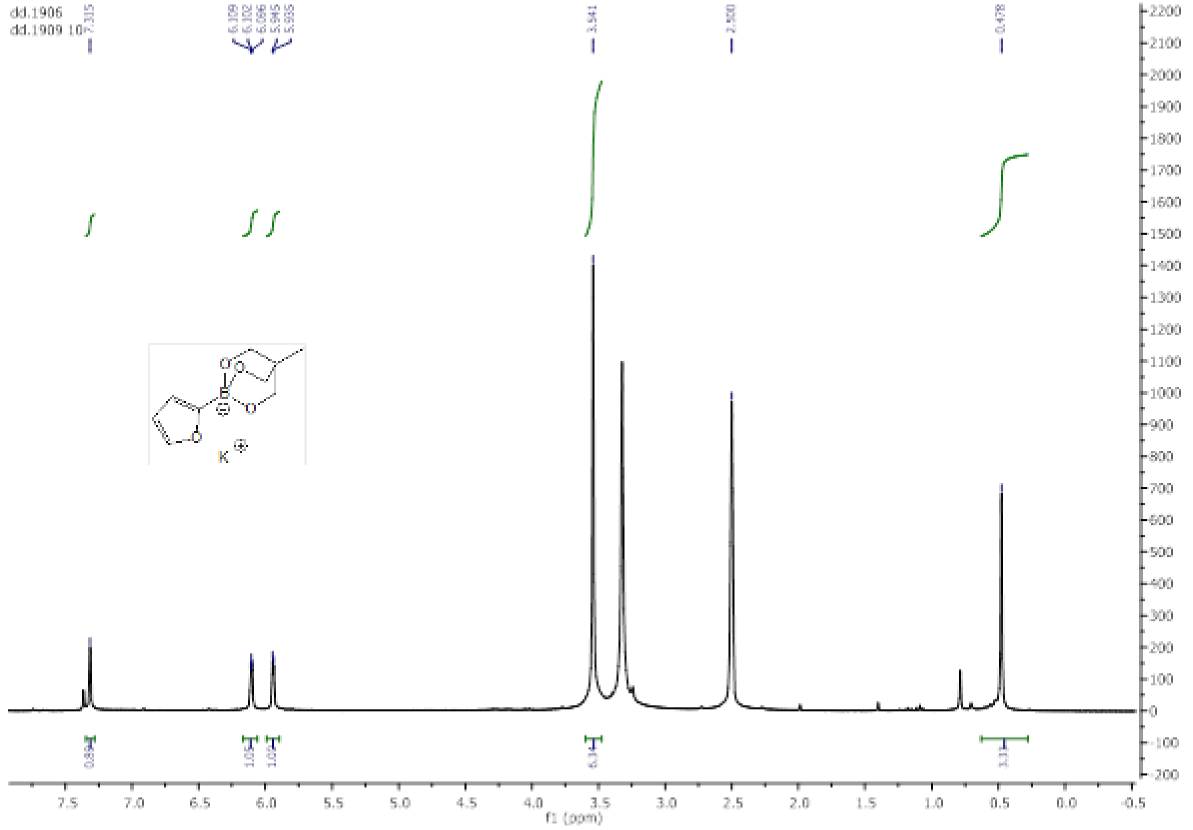

13C NMR **2i**

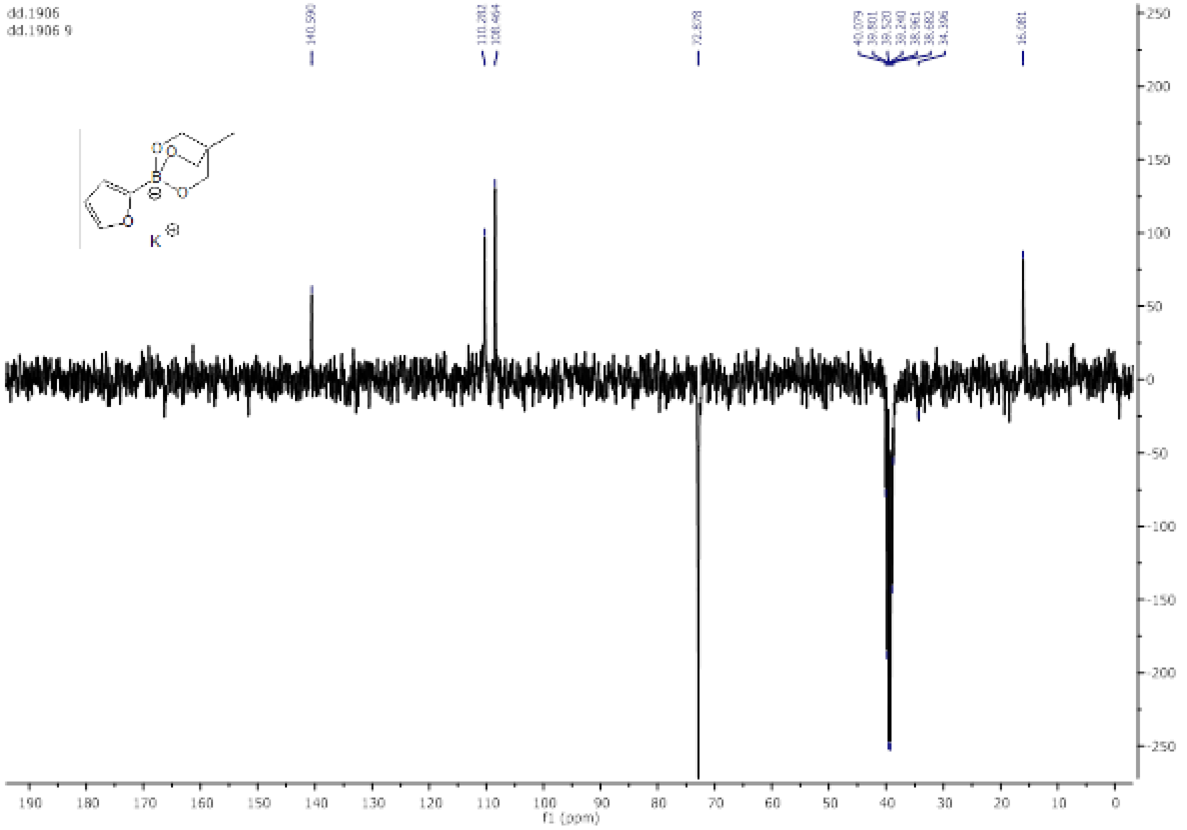

^{1}H NMR of **2j**

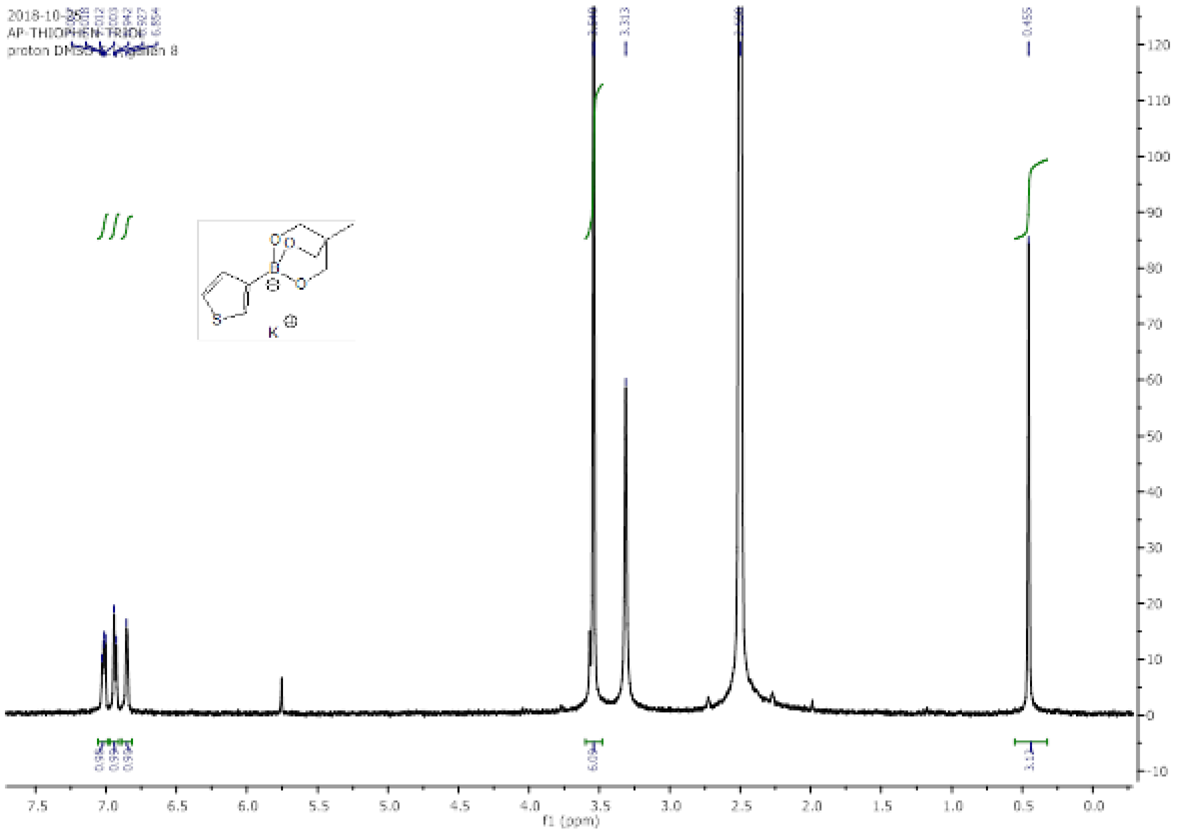

^{13}C NMR of **2j**

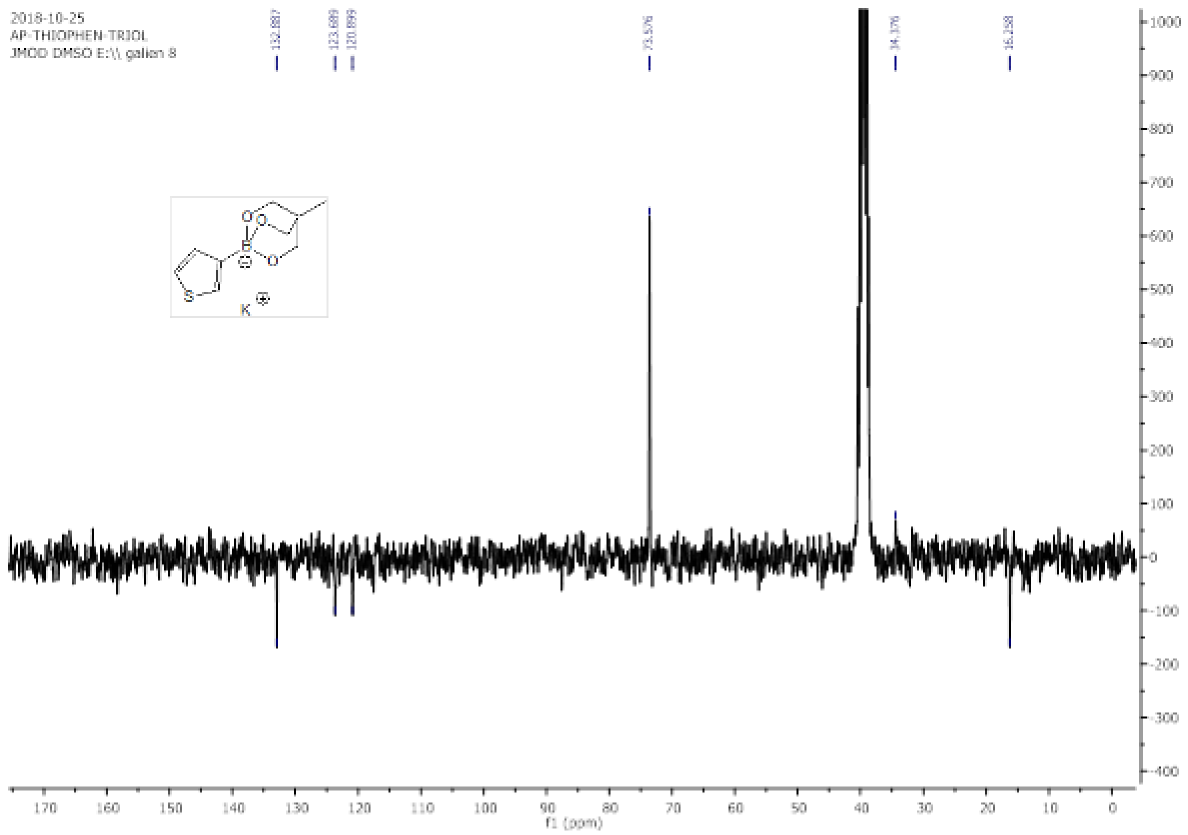

^{1}H NMR **3a**

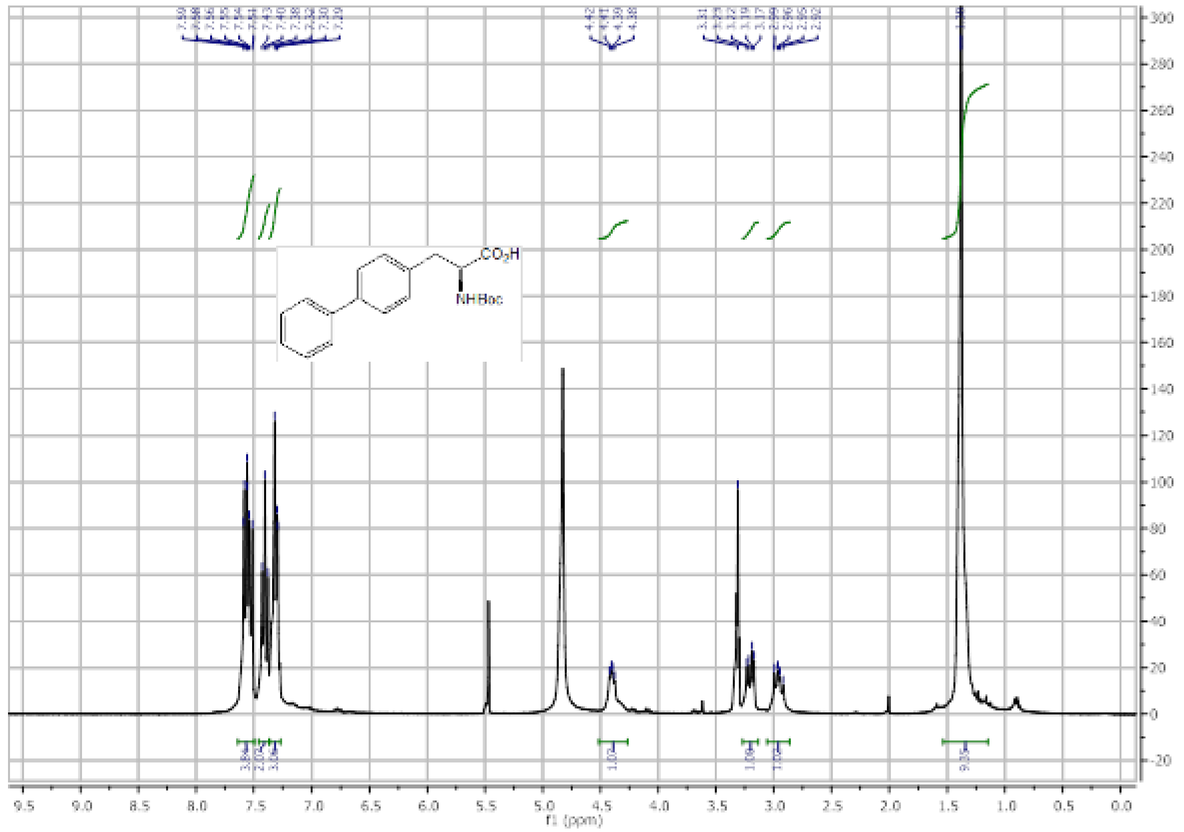

13C NMR 3a

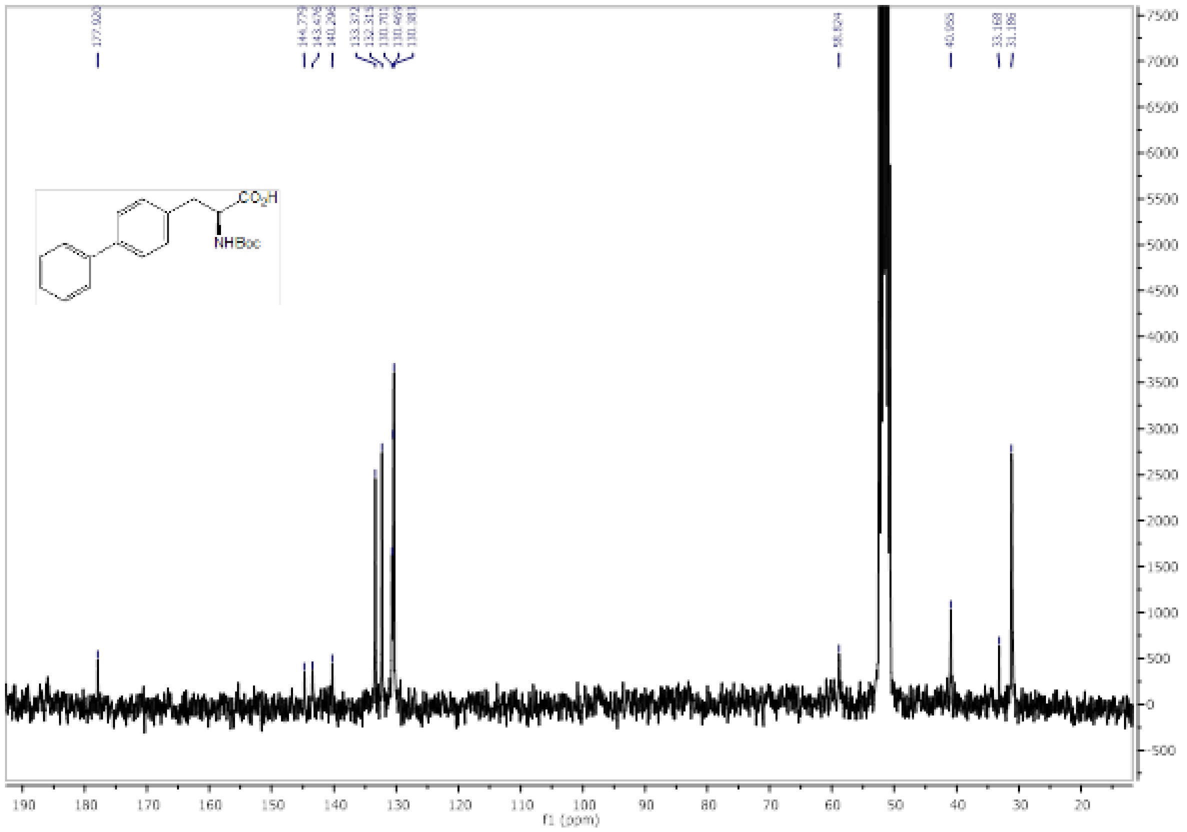

^{1}H NMR of **3d**

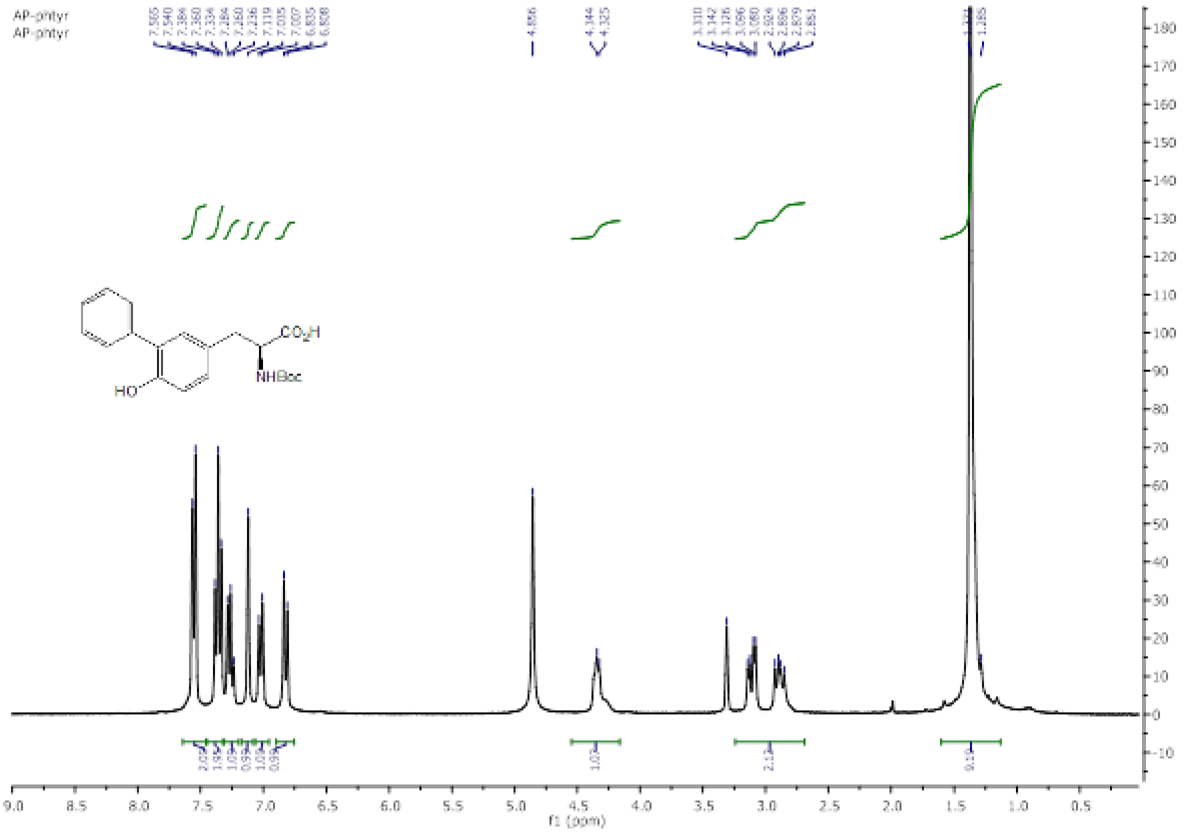

^{13}C NMR of **3d**

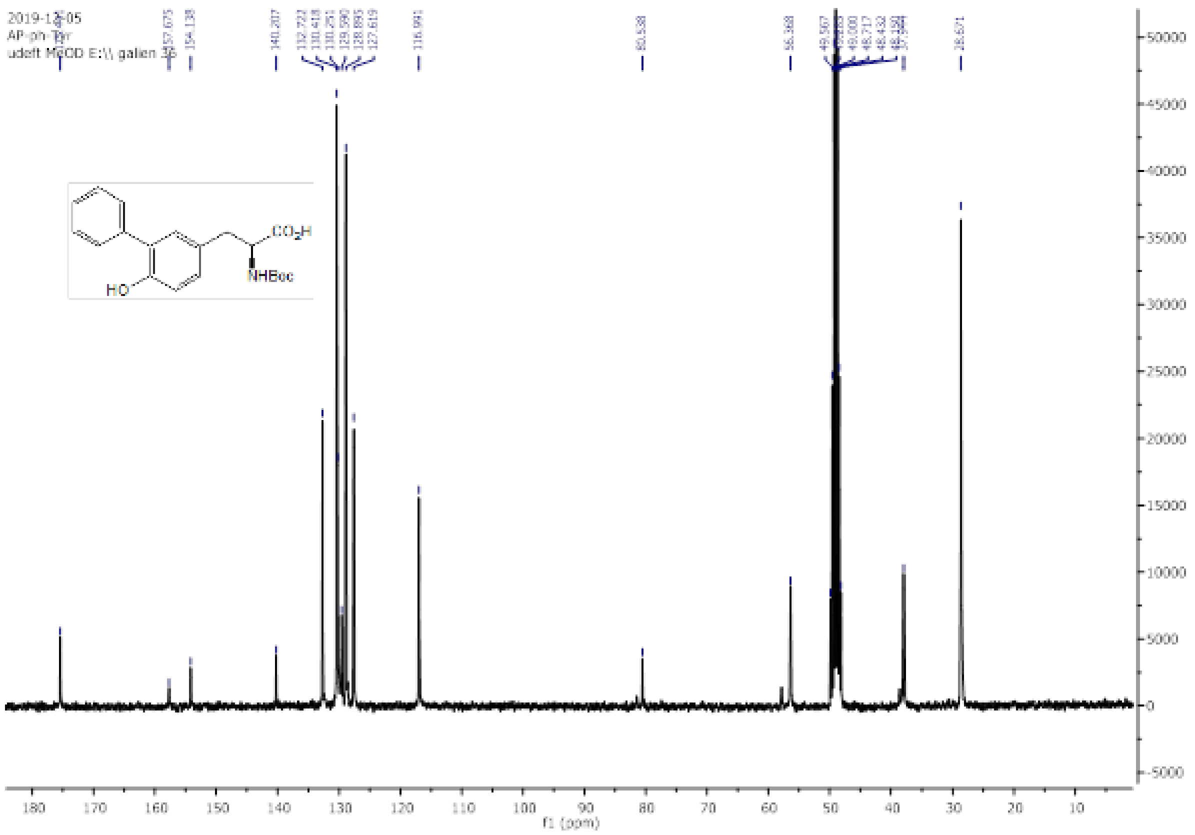

^{1}H NMR **3e**

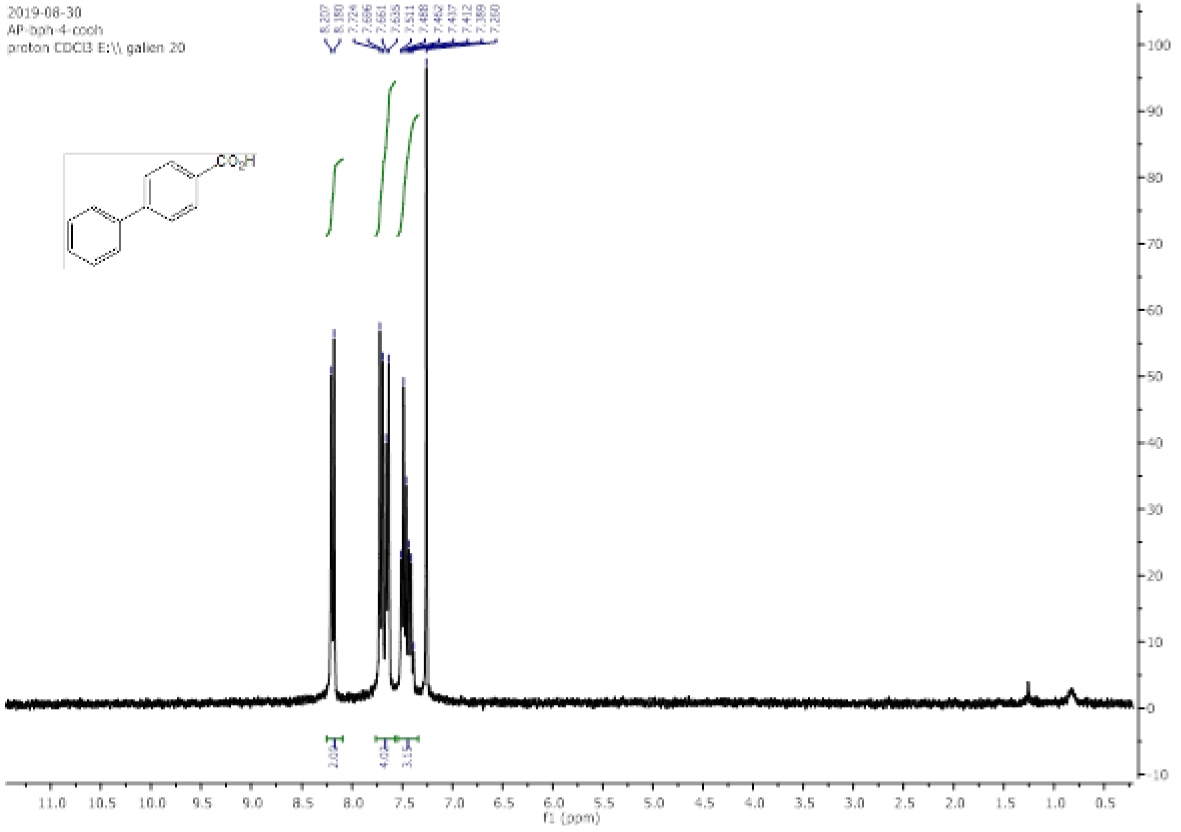

13C NMR **3e**

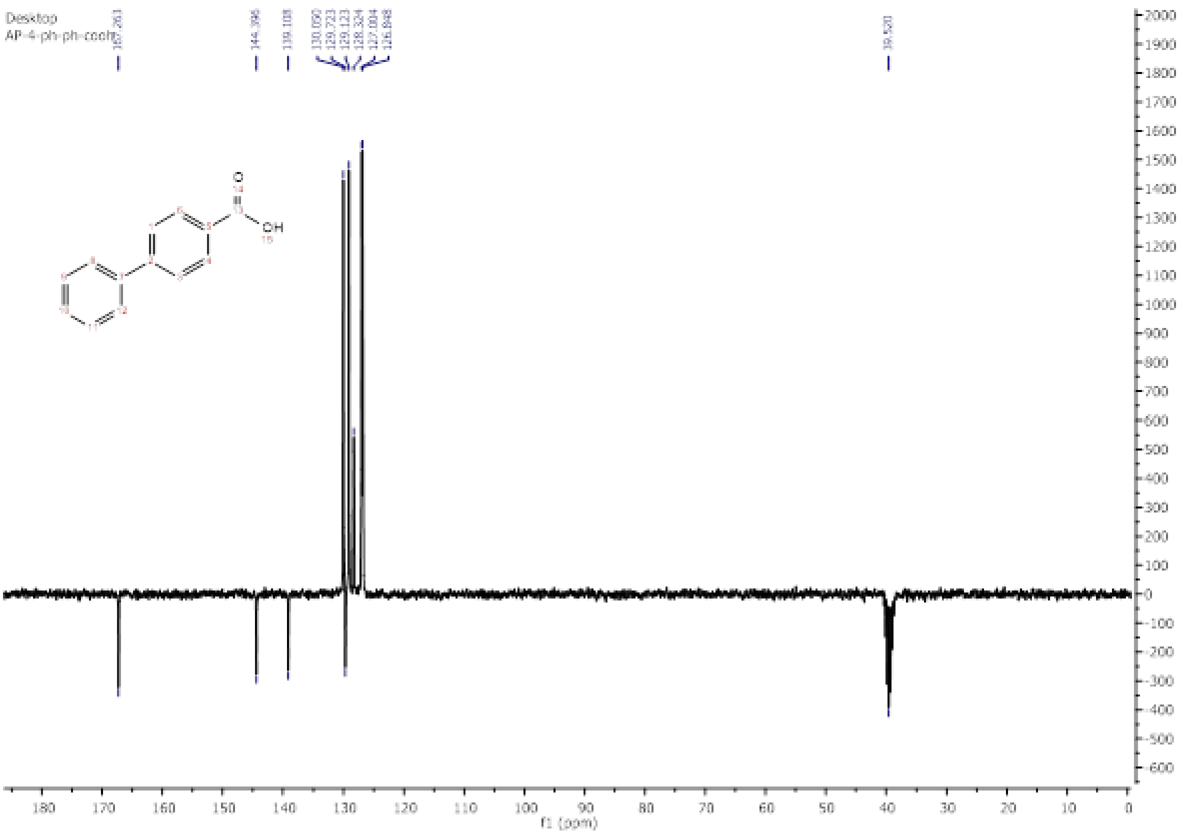

1H NMR 3f

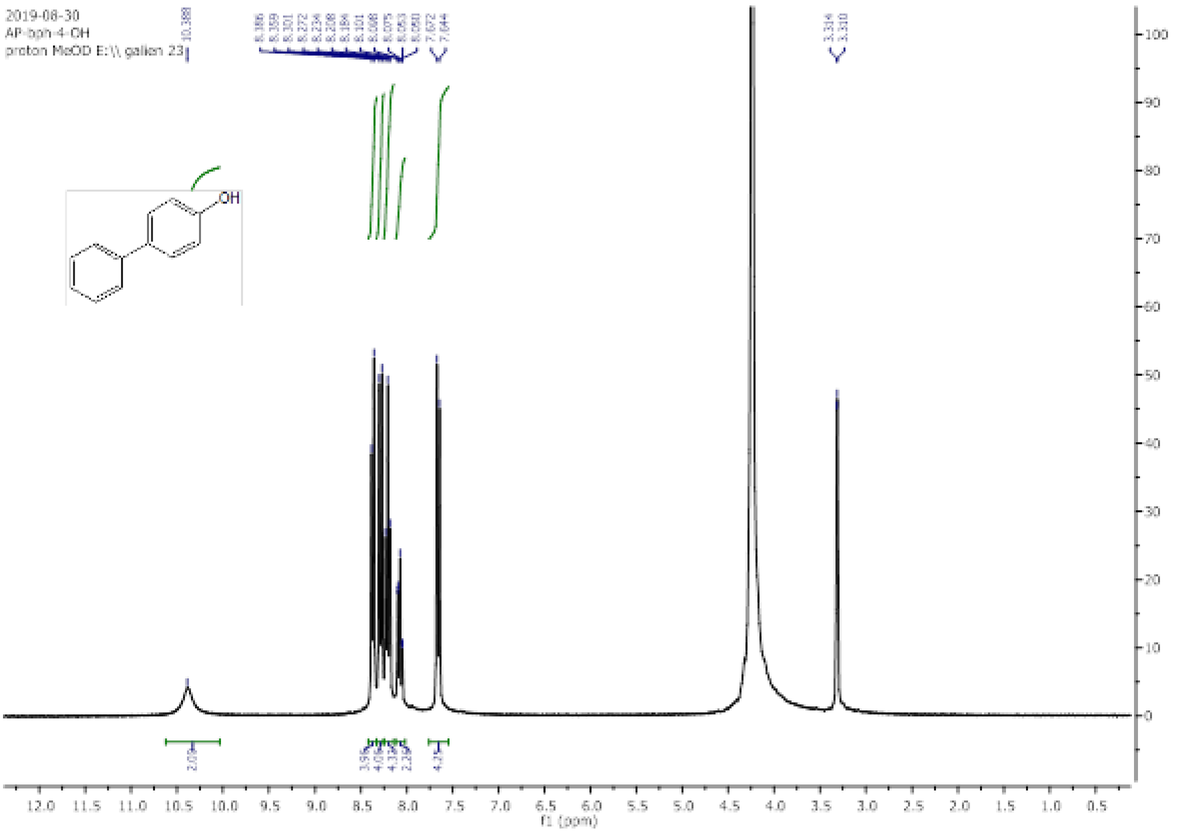

13C NMR 3f

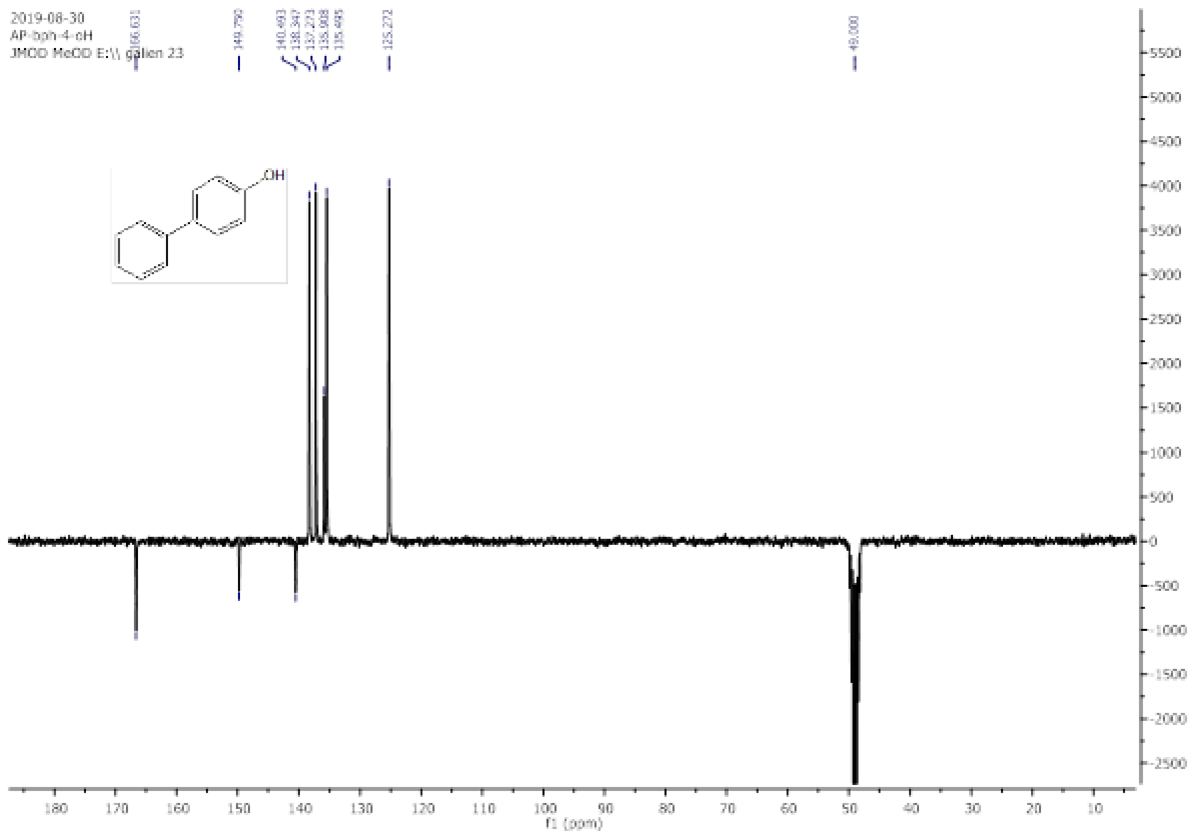

^{1}H NMR **3h**

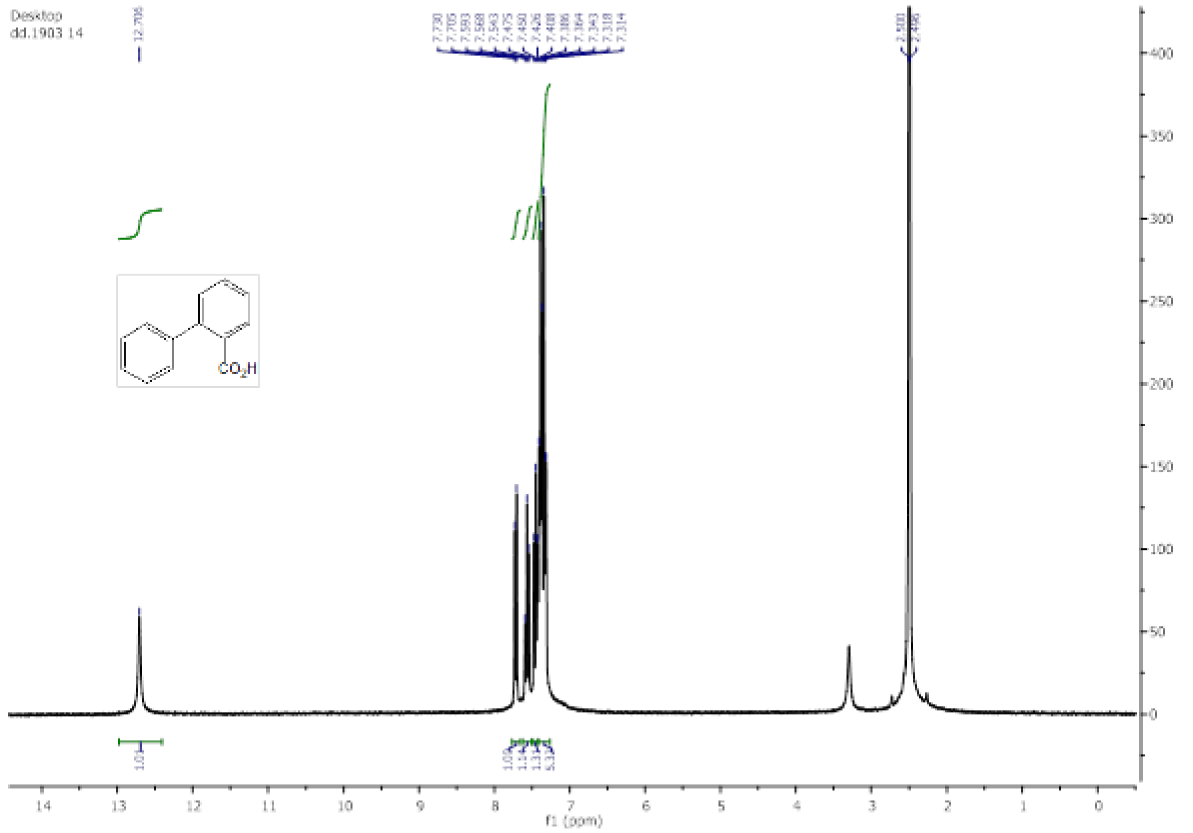

^{13}C NMR **3h**

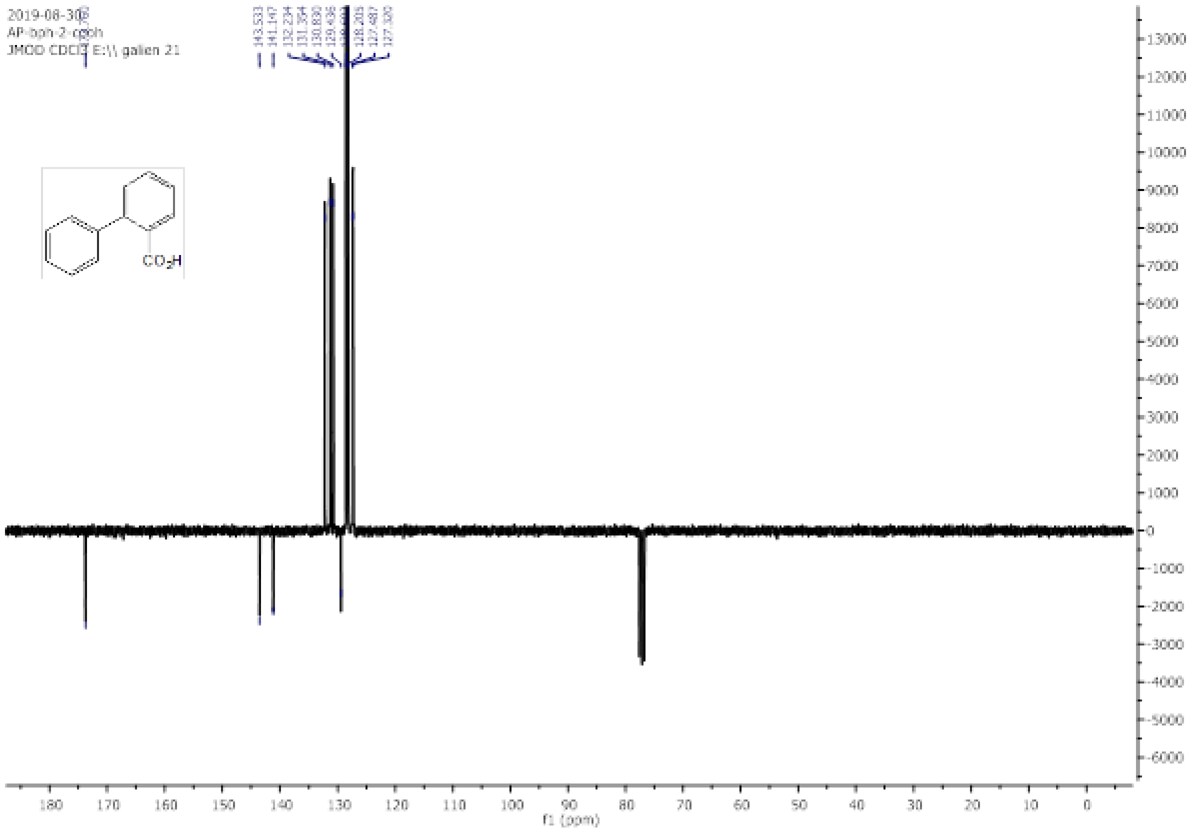

^{1}H NMR **3i**

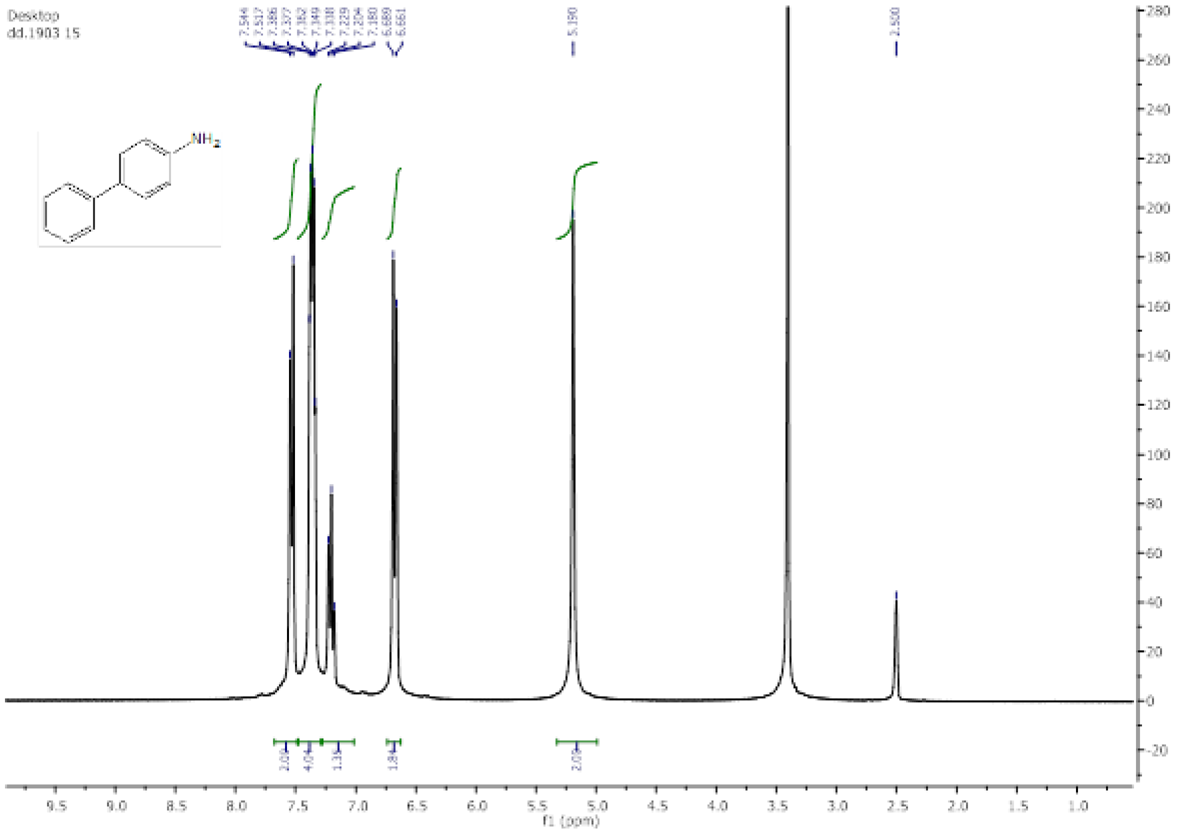

^{13}C NMR **3i**

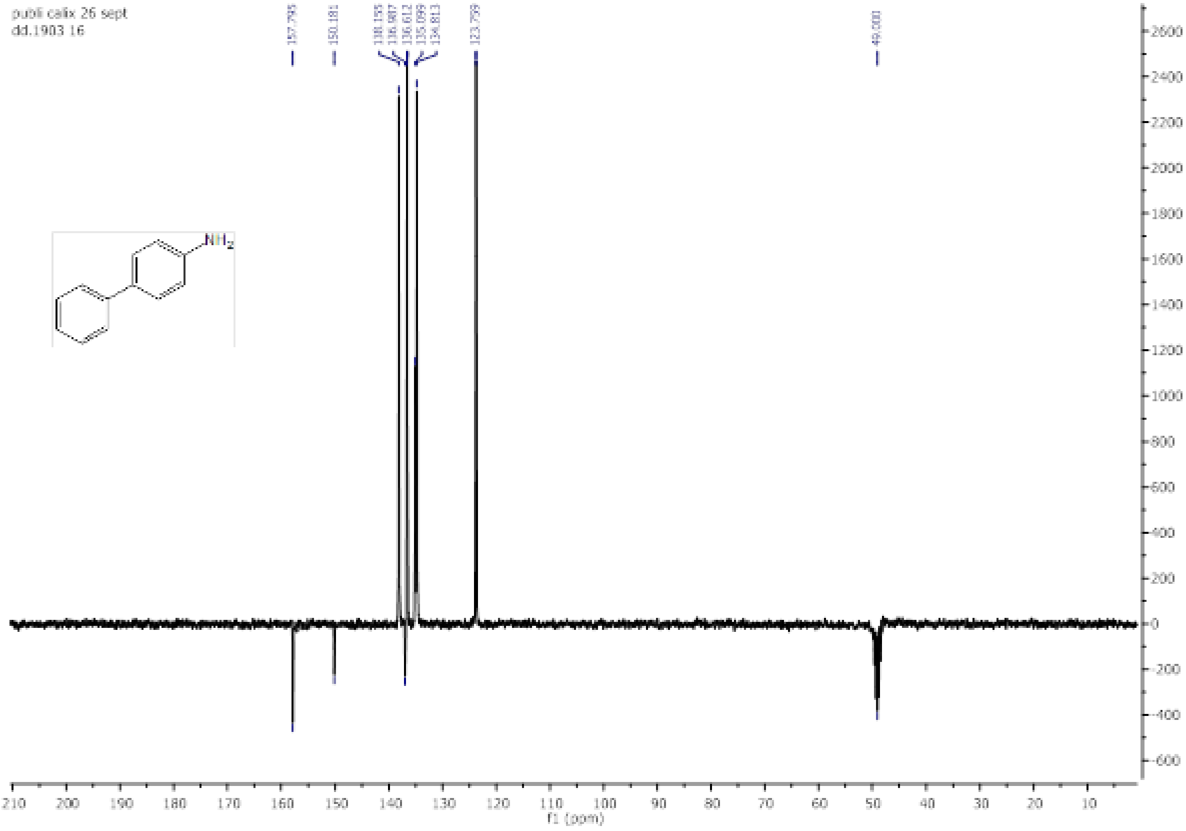

^{1}H NMR **3j**

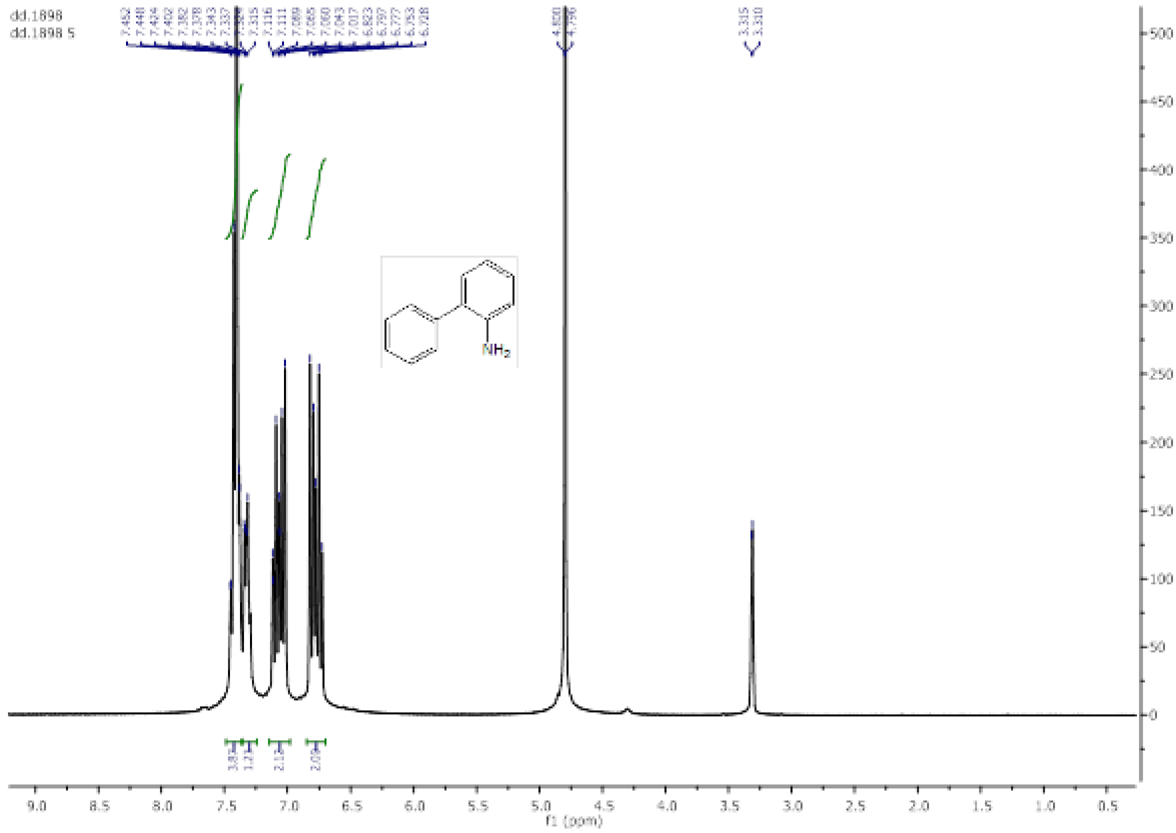

^{13}C NMR **3j**

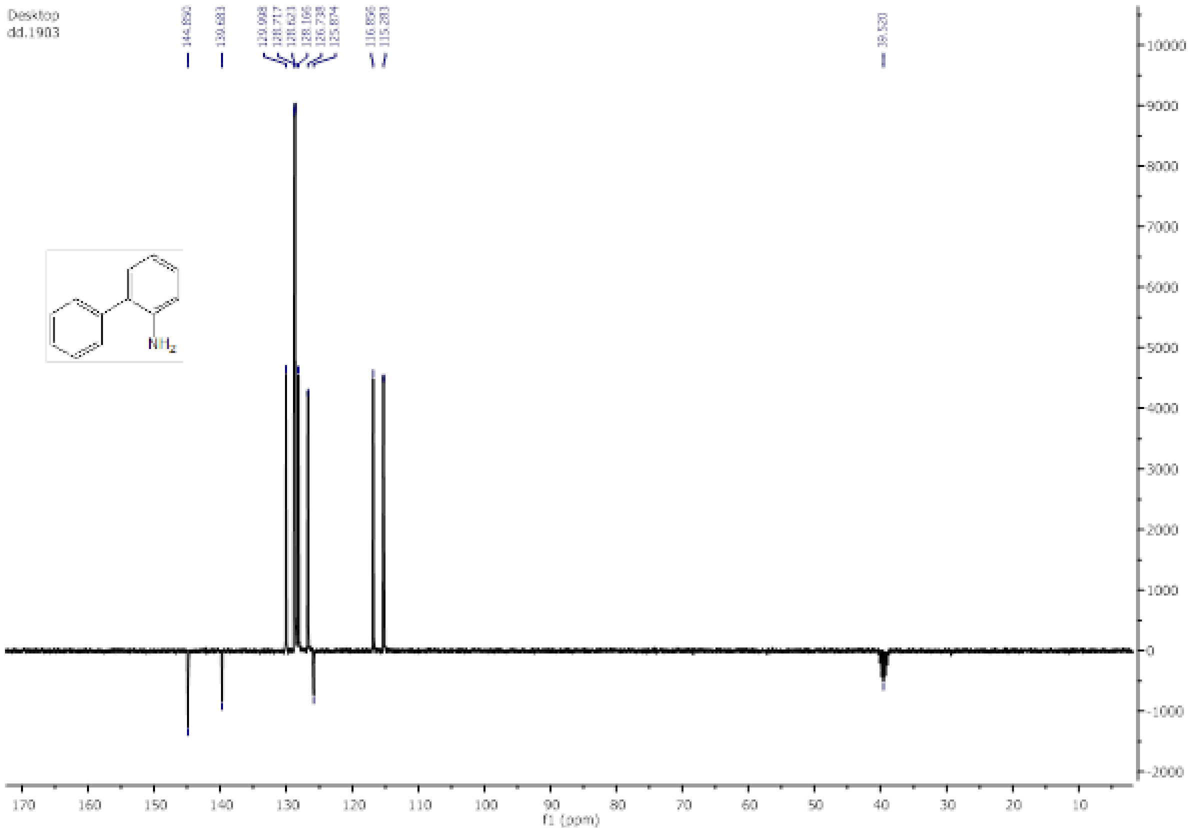

^{1}H NMR **3af**

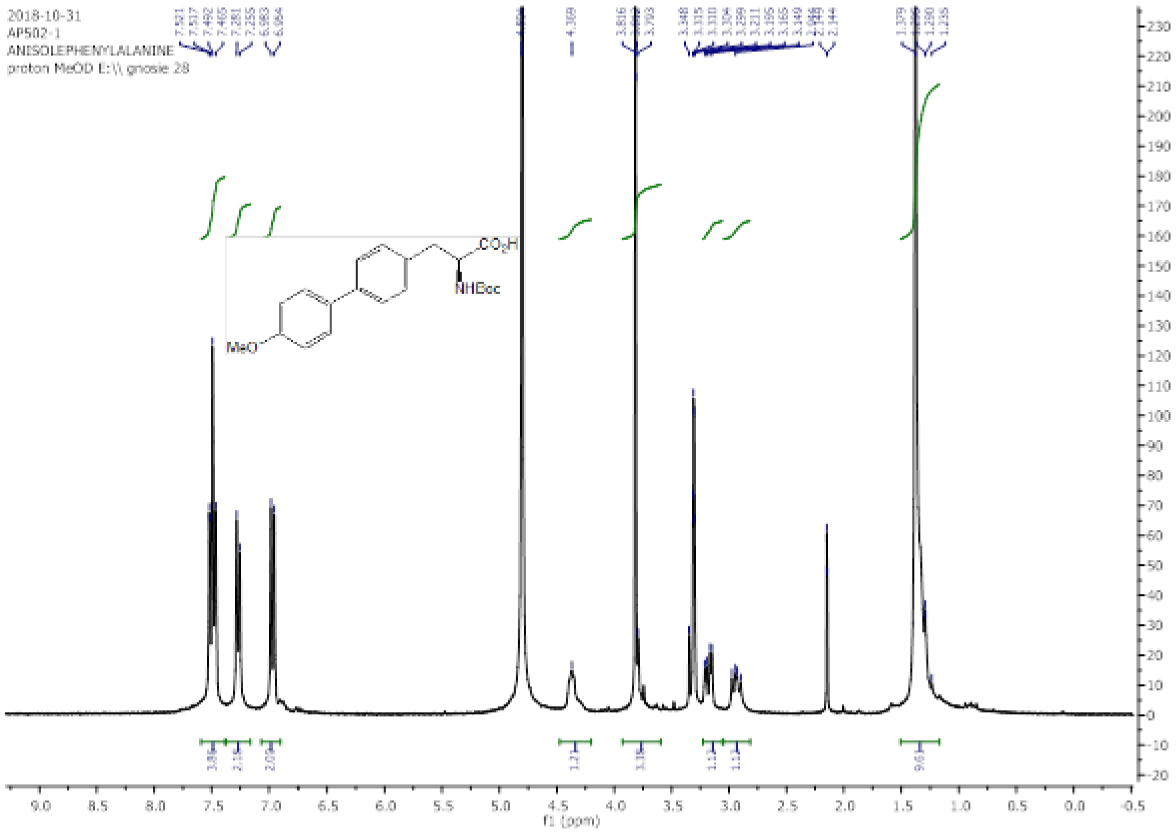

^{13}C NMR **3af**

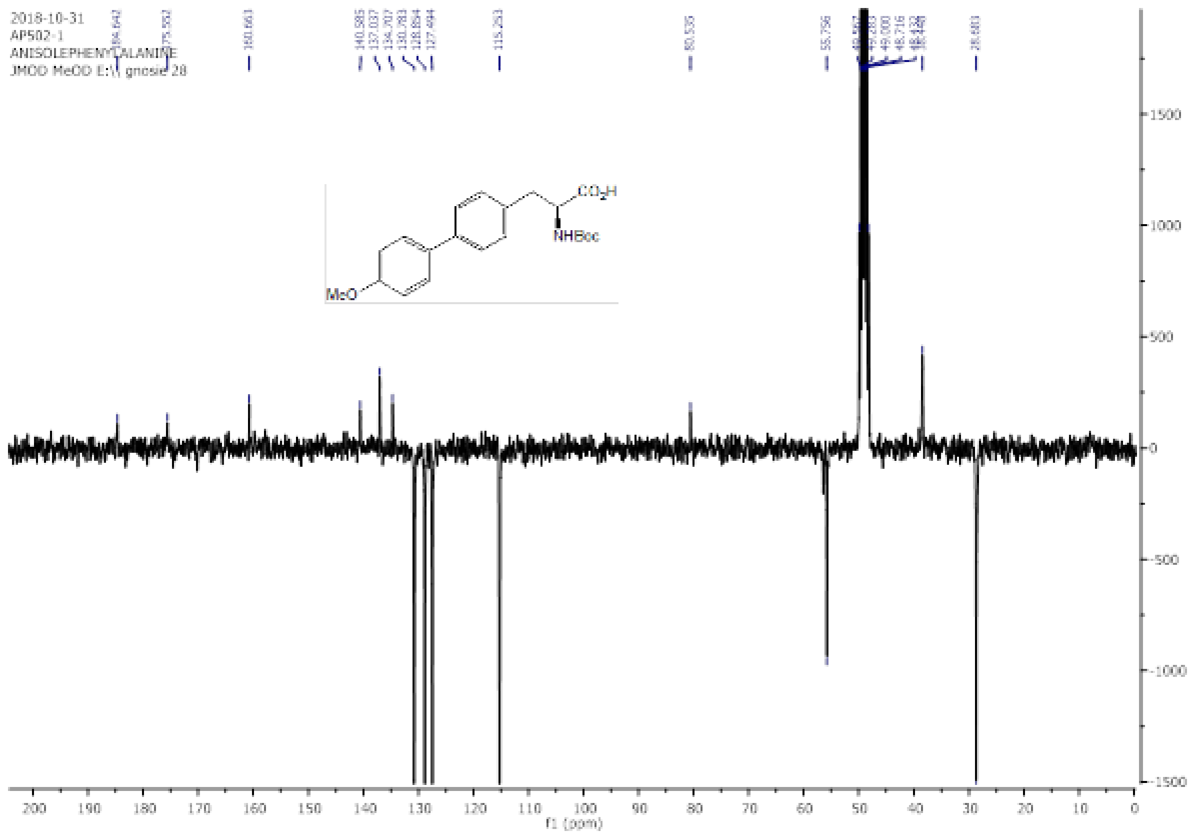

^{1}H NMR **3ag**

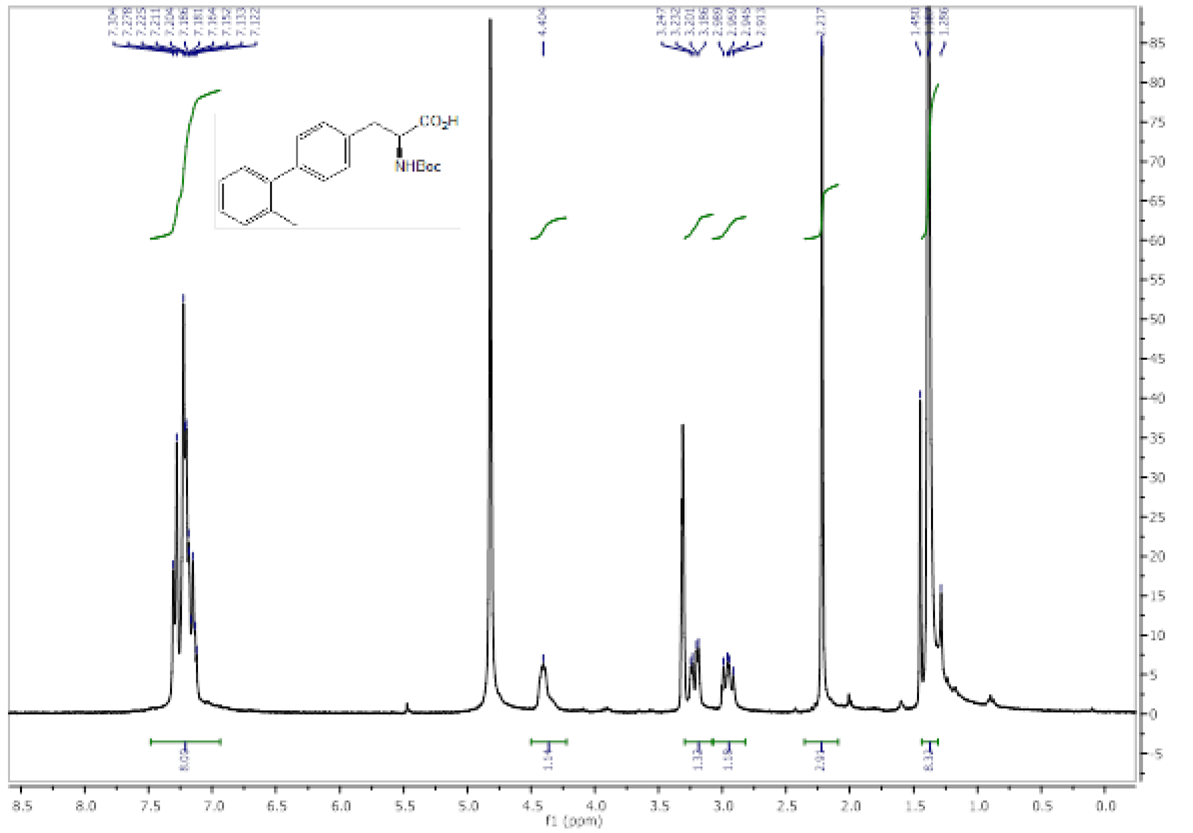

^{13}C NMR **3a**g

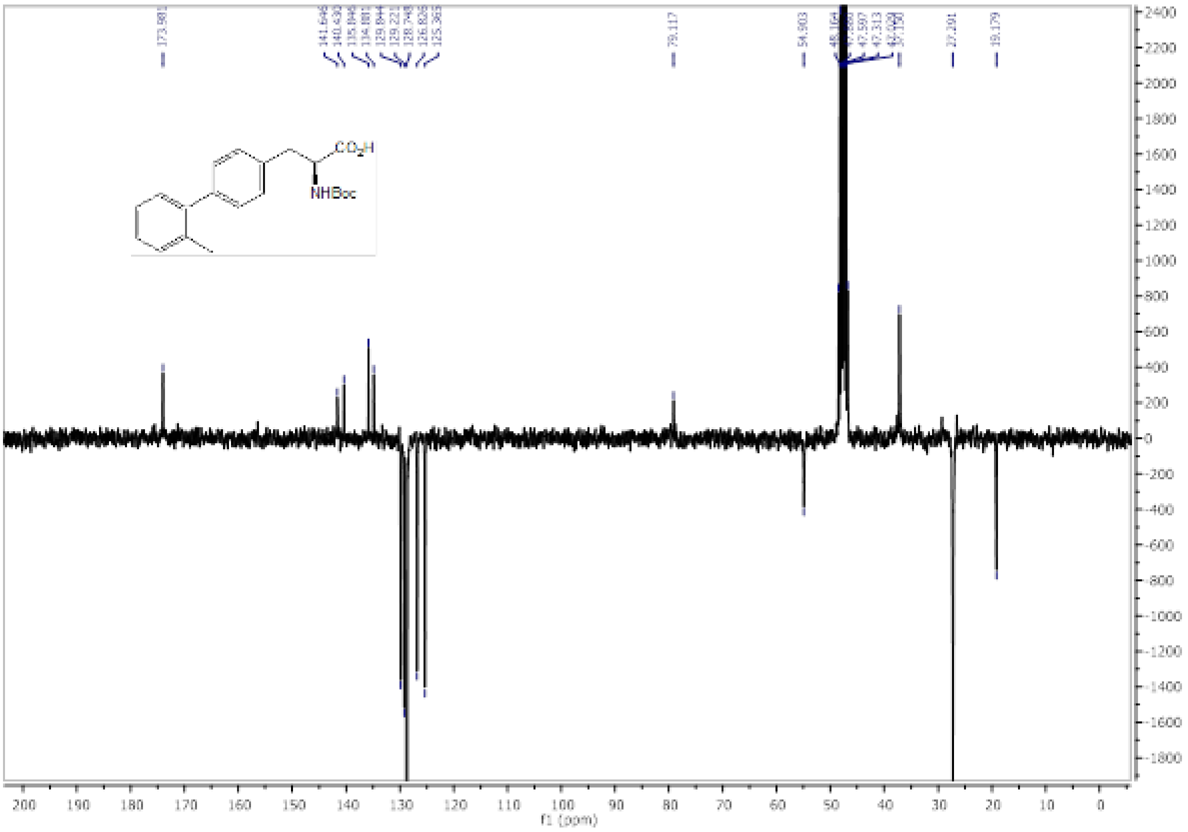

^{1}H NMR **3ah**

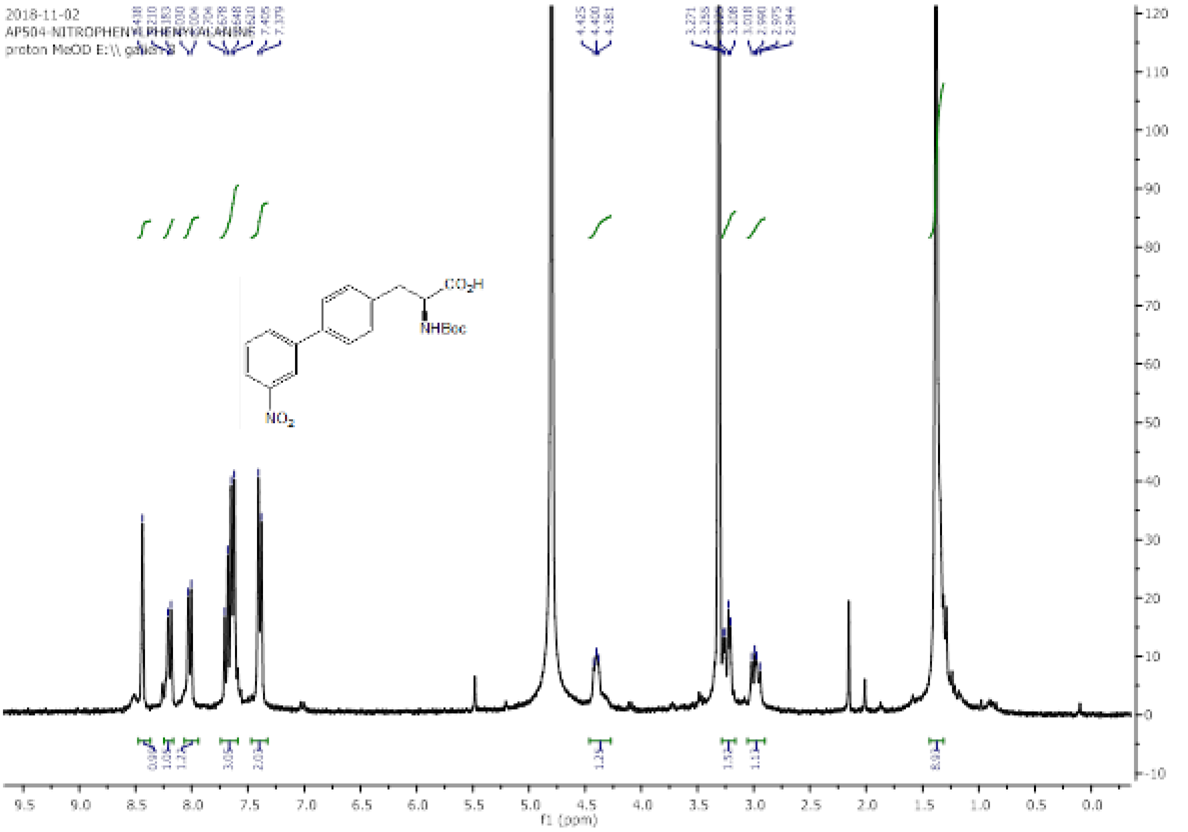

^{13}C NMR **3ah**

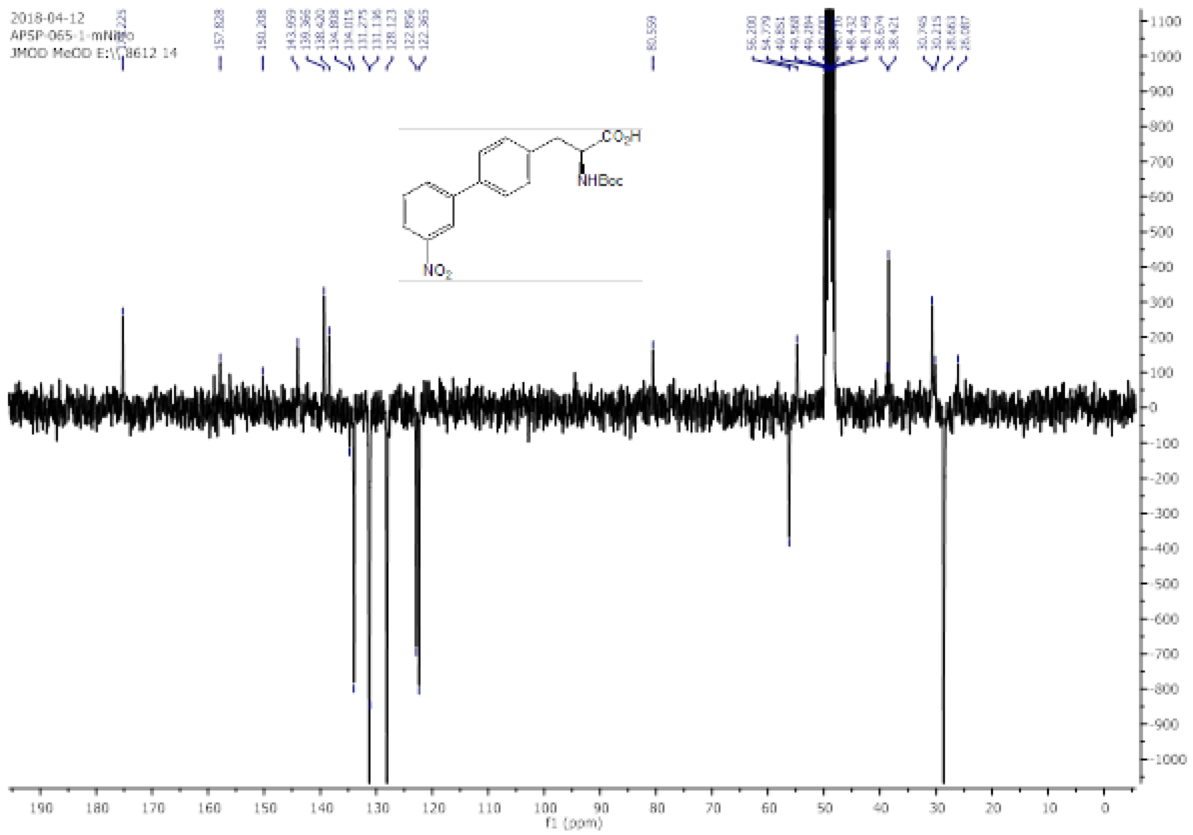

^{1}H NMR **3ai**

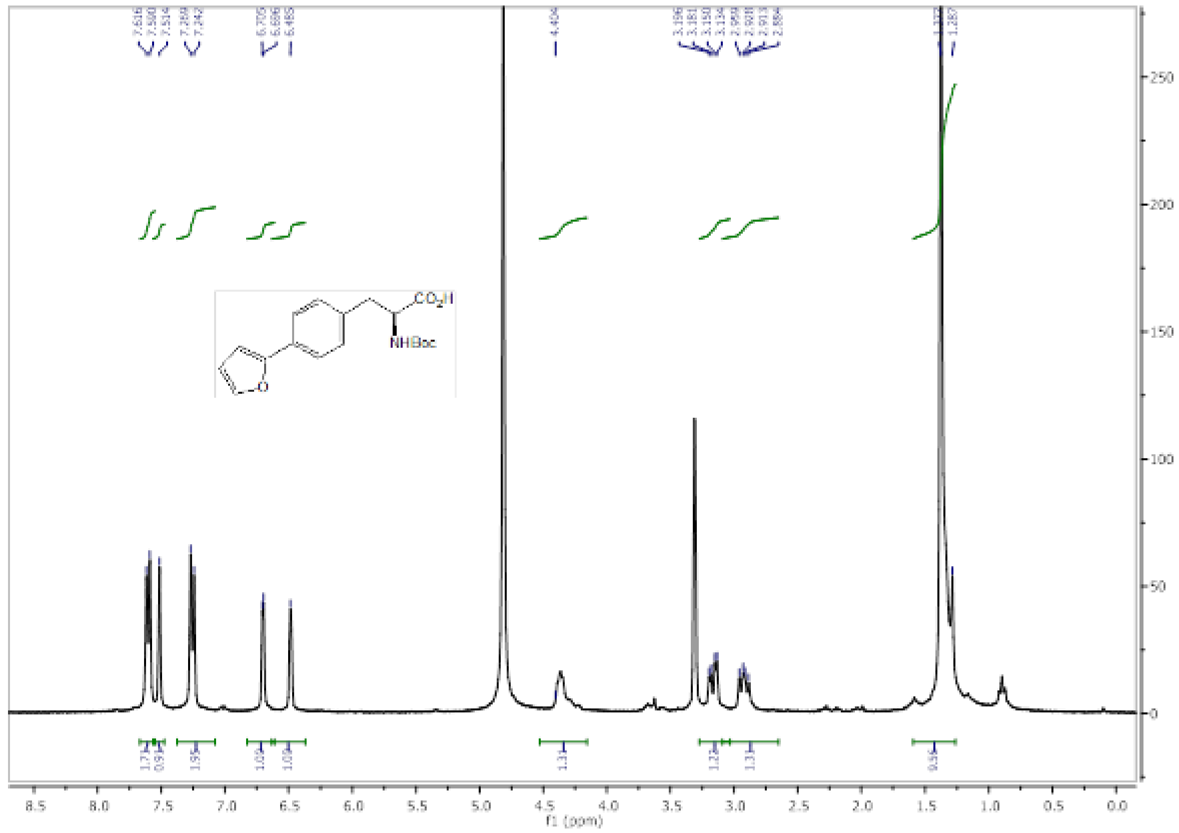

^{13}C NMR **3ai**

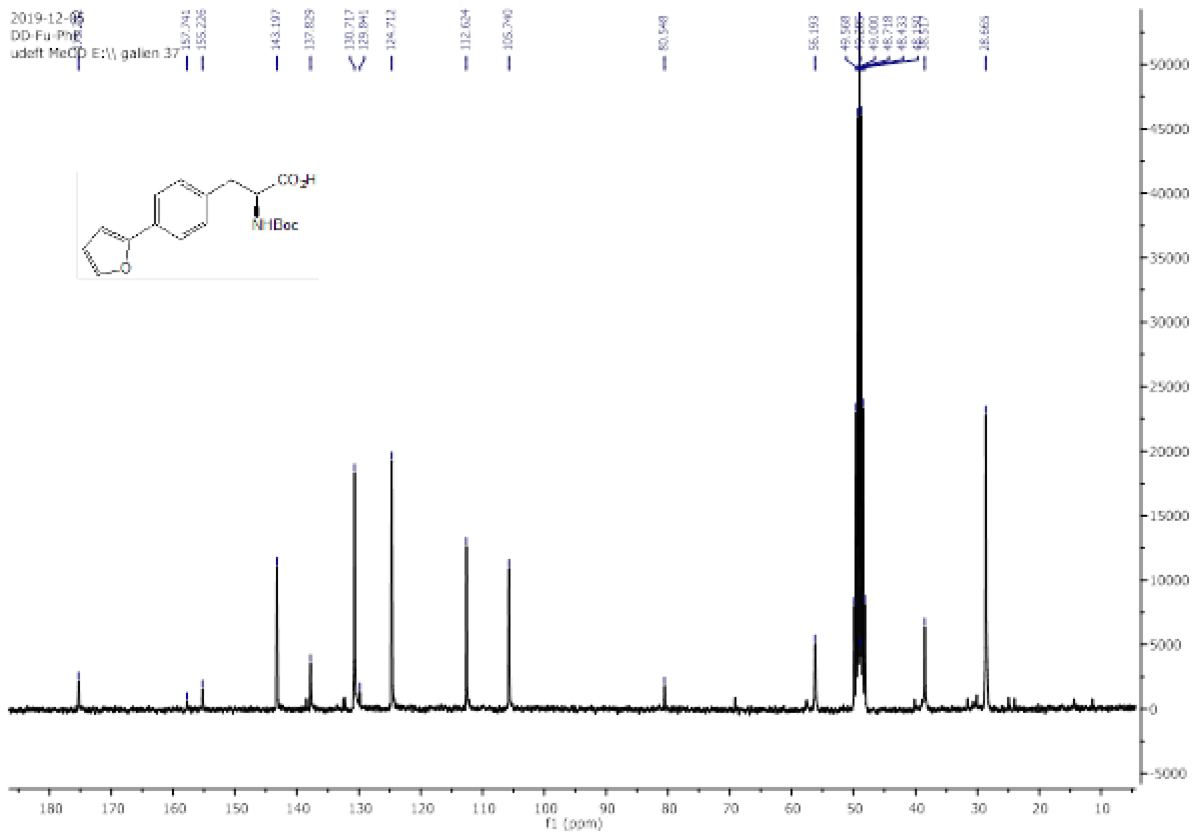

^{1}H NMR **3aj**

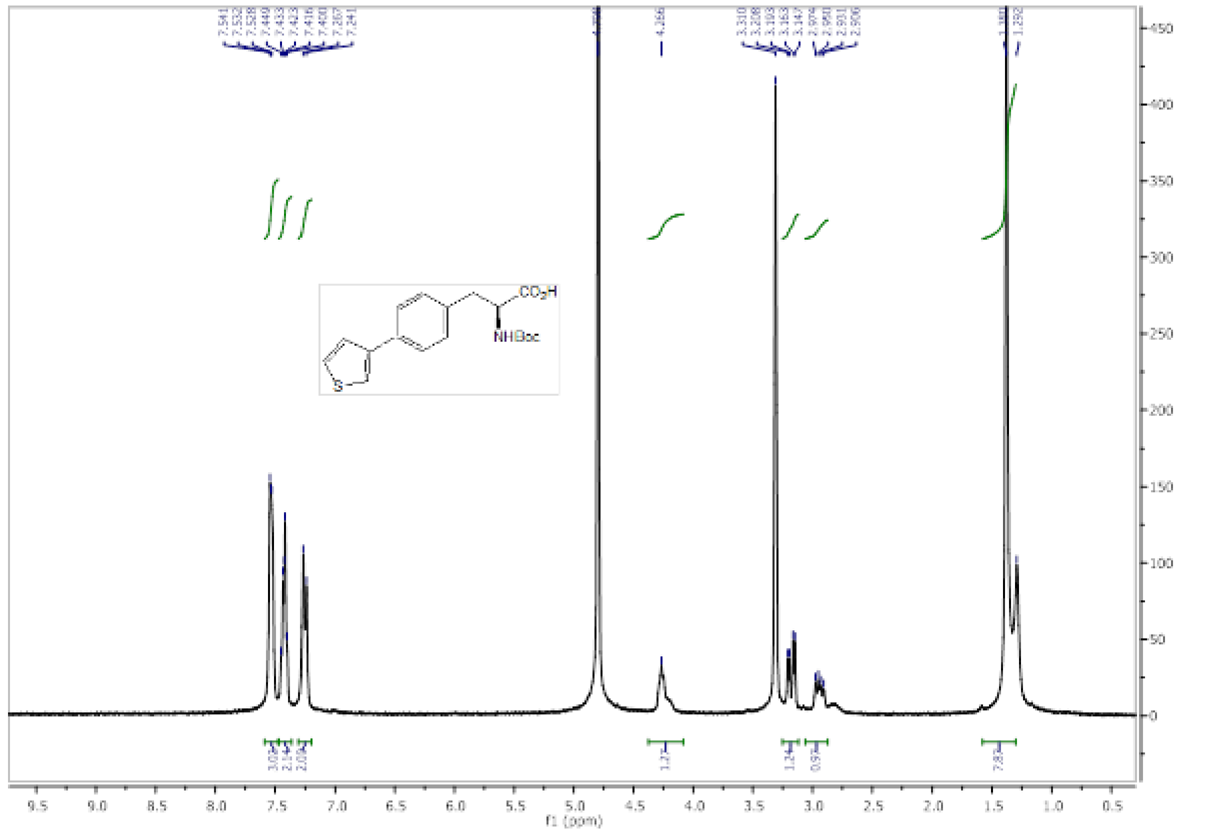

^{13}C NMR **3aj**

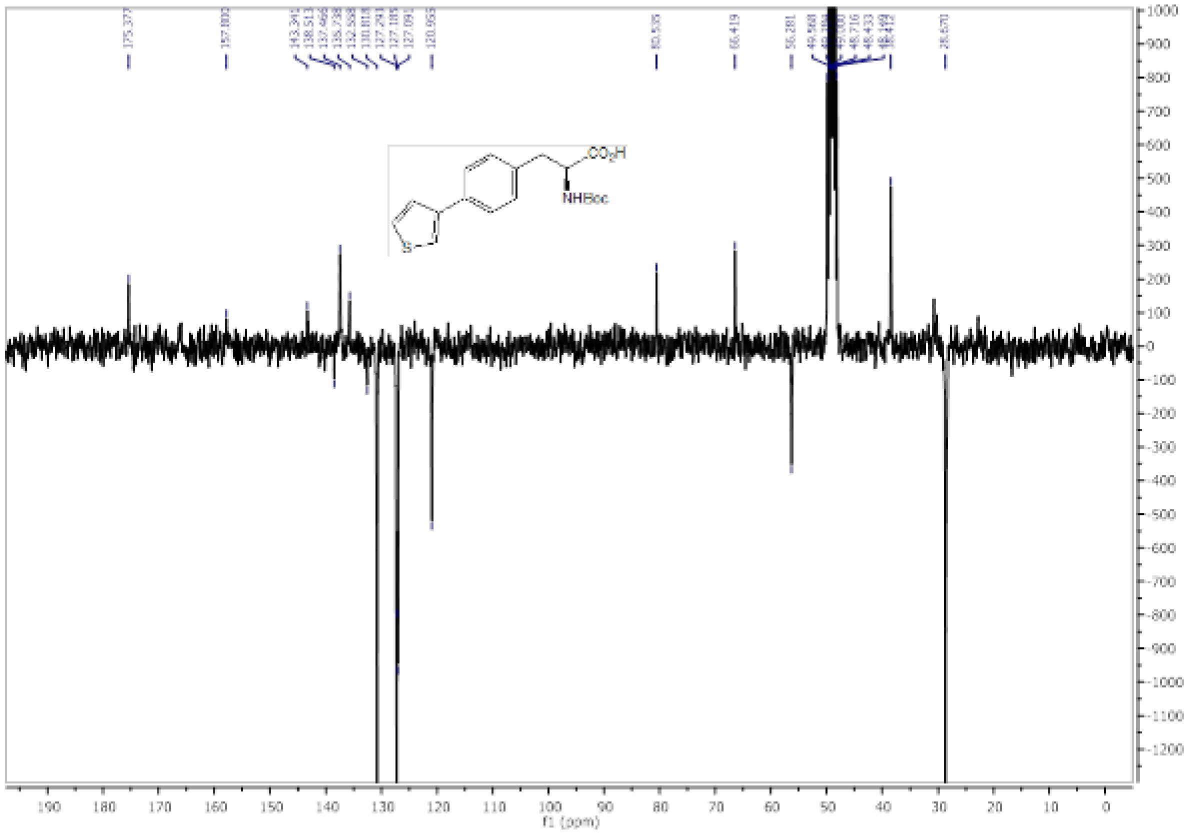
175.377
143.341
138.513
137.466
120.953
80.535
66.419
56.281
48.716
48.433
38.412
28.670
CO2H
NHBoc
S
f1 (ppm)
